# Triiodothyronine for the treatment of critically ill patients with COVID-19 infection: A structured summary of a study protocol for a randomised controlled trial

**DOI:** 10.1186/s13063-020-04474-0

**Published:** 2020-06-26

**Authors:** Constantinos Pantos, Georgia Kostopanagiotou, Apostolos Armaganidis, Athanasios Trikas, Ioulia Tseti, Iordanis Mourouzis

**Affiliations:** 1 Department of Pharmacology, Medical School, National and Kapodistrian University of Athens, 75 Mikras Asias Ave.,11527 Goudi, Athens, Greece; 2 Second Department of Anesthesiology, Medical School, National and Kapodistrian University of Athens, Attikon University Hospital, Athens, Greece; 3 2nd Department of Critical Care, “Attikon” University Hospital, Medical School, National and Kapodistrian University of Athens, Athens, Greece

**Keywords:** COVID-19, Randomised controlled trial, protocol, thyroid hormone, triiodothyronine, hypoxia, multi-organ dysfunction, viral infection

## Abstract

**Objectives:**

Tissue hypoxia is the main cause of multi-organ dysfunction in sepsis. However, effective pharmacological treatments to combat sepsis-induced tissue hypoxia are not available. Emerging experimental and clinical evidence reveals an evolutionary conserved action of thyroid hormone (TH) to adapt injured tissue to hypoxic conditions via its action on p38 MAPK, Akt signaling pathways. In addition, TH has favorable effects on the immune system and viral load in infected tissue. Non-Thyroid Illness Syndrome is common in sepsis, acute myocardial infarction and trauma and is associated with increased mortality. Thus, TH may be a novel treatment in the setting of critical illness due to viral infection in which hypoxia prevails. The present study aims to address the efficacy and safety of acute administration of triiodothyronine (T3) in critically ill COVID-19 infected patients requiring mechanical respiratory support or Extra Corporeal Membrane Oxygenation (ECMO).

**Trial design:**

This study is a phase II, parallel, 2-arm (1:1 ratio), multi-centre, prospective, randomized, double-blind, placebo controlled trial.

**Participants:**

Male and female patients aged over 18 years old who are diagnosed with pulmonary infection due to COVID-19, admitted to Intensive Care Unit and requiring mechanical ventilation or ECMO will be enrolled in this trial. Patients will be excluded in cases of pregnancy, severe systemic disease with life expectancy less than 6 months, participation in another trial of an investigational drug or device, corticosteroid and/or sympathomimetic use before initiation of treatment. All data will be collected in electronic CRF files. Participants will start to be recruited from the ICU center of “ATTIKO” University Hospital in Greece. We aim to include two more clinical sites in the trial one from Greece and one from Germany

**Intervention and comparator:**

Intervention: T3 Solution for injection 10 μg/ml. The dose administered will be 0.8g/kg i.v. bolus and will be followed by an infusion of 0.113g. kg-1.h-1 i.v. for 48 hours (therapeutic dose). After the first 48h, a maintenance dose will be administered corresponding to 50% of the therapeutic dose (0.057g. kg-1.h-1 i.v.). Drug administration will stop after successful weaning or end of follow up (maximum 30 days).

Comparator: Placebo with composition and dosage identical apart from the active substance.

**Main outcomes:**

The primary outcome assessed in the present study will be the percentage of patients successfully weaned after 30 days of follow-up. Successful weaning is defined as no requirement for ventilatory support after extubation (mechanical support) or support from ECMO for 48 hours.

**Randomisation:**

An allocation sequence to one of the groups will be prepared by the Sponsor of the study. A 1:1 treatment allocation will be adopted. An electronic CRF will be used incorporating IWRS in order to assure proper randomization and unblinding in emergency cases. The representative of the sponsor will get a copy of randomization codes. The information of the randomization codes will then be locked in the database until the time at which an interim analysis or final analysis is performed.

**Blinding (masking):**

Participants, caregivers, and all investigators assessing the outcomes will be blinded to group assignment.

**Numbers to be randomised (sample size):**

The sample size of 60 patients (that indicates 30 subjects for each group) will have 84% power to detect the estimated difference between the two study groups. The criterion for significance (alpha) has been set at 0.05 and the test is 2-tailed.

**Trial Status:**

Protocol number T3inj-02/ThySupport, version 03, May 11, 2020. The trial is not recruiting yet. The trial will start recruitment June 18^th^ 2020. Estimated recruitment will finish June 18^th^, 2021.

**Trial registration:**

Triiodothyronine for the Treatment of Critically Ill Patients With COVID-19 Infection (Thy-Support), ClinicalTrials.gov Identifier: NCT04348513, date of trial registration: April 16, 2020, EudraCT Identifier: 2020-001623-13, date of trial registration: April 22, 2020

**Full protocol:**

The full protocol is attached as an additional file, accessible from the Trials website (Additional file 1). In the interest in expediting dissemination of this material, the familiar formatting has been eliminated; this Letter serves as a summary of the key elements of the full protocol.

## Supplementary information

**Additional file 1.** Full study protocol.

## Data Availability

The Data Monitoring Committee will have access to the final trial dataset. The data will be available from the corresponding author on reasonable request and upon permission from the sponsor of the study.

