# Supplementary material for: Triiodothyronine for the treatment of critically ill patients with COVID-19 infection: A structured summary of a study protocol for a randomised controlled trial
Source: Trials. 2020 Jun 26;21:573. doi: 10.1186/s13063-020-04474-0 (PMC7316579; doi:10.1186/s13063-020-04474-0)
Supplement: Supplementary file 1 — Additional file 1. Full study protocol. [file 13063_2020_4474_MOESM1_ESM.docx]

**1. SUMMARY**

**Study title and code:**

Triiodothyronine (T3) for the treatment of critically ill patients with COVID-19 infection ***("ThySupport")***

**EudraCT No:** 2020-001623-13

**Study type and design:** This study is a phase II, parallel, prospective, randomized, double-blind, placebo controlled trial.

**Study aim:** The present study aims to address the efficacy and safety of acute administration of T3 on ICU patients diagnosed with pulmonary infection due to COVID-19 that require mechanical respiratory support or ECMO.

**Study treatment:** Intravenous T3 administration in ICU patients *diagnosed with pulmonary infection due to COVID-19*.

**Number and characteristics of patients:** 60 ICU patients (Age> 18 years old) diagnosed with pulmonary infection due to COVID-19 requiring mechanical respiratory support or ECMO will be included in the study.

**Study duration:** The study will start at June 2020 and is programmed to be completed by June 2021. Treatment period will last till successful weaning or end of follow-up. Follow up will be performed for 1 month.

**Objectives:** The ***primary objective*** of the study is to determine whether the administration of intravenous T3 in ICU patients *diagnosed with pulmonary infection due to COVID-19* facilitates weaning from cardiorespiratory support compared to placebo.

**Main efficacy criteria:** The primary end-point assessed in the present study will be the percentage of patients successfully weaned after 30 days of follow-up after intubation. Successful weaning is expected in 15% of patients at 30 days in placebo group. We assume that T3 treatment can increase successful weaning to 50% of patients which is a significant clinical improvement in prognosis.

**Safety criteria:** The following safety criteria will be assessed: cardiac and non-cardiac death, cardiac arrest, electromechanical dissociation, pulmonary embolism, new myocardial infarction, stroke, pulmonary oedema, cardiogenic shock and hypotension, septic shock, serious bleeding, episodes of arrhythmias (paroxysmal supraventricular tachycardia, atrial fibrillation, sustained ventricular tachycardia). Any iindividual adverse event (AE) will be evaluated by the investigators and they will be reported to the sponsor and Coordinator during the duration of the study.

**Number of Centres and Country(ies):** This study will start in ICU center of “ATTIKO” University Hospital. We aim to include two more clinical sites in the trial one from Greece and one from Germany.

**Sponsor statement:**

### The study will be conducted according to this protocol, the Good Clinical Practice (GCP), the ICH guidelines, local laws and obligations and the World Medical Association Declaration of Helsinki *(Appendix 1)*.

**2. BACKGROUND AND RATIONALE**

**Background**

**Introduction**

Covid-19 has caused a pandemic with increased mortality in the population particularly in aging people and patients with chronic disease. A number of infected patients require hospital care and severe cases are admitted to ICU (intensive care units) due to sepsis/septic shock. The mortality rate of patients requiring ICU treatment was reported to be between 81%-97% (1, 2).

Sepsis is a complex disorder that develops as deregulated host response to an infection and is associated with acute organ dysfunction and a high risk of death.

The world health assembly and WHO made sepsis a global health priority in 2017 and have adopted a resolution to improve the prevention , diagnosis and management of sepsis. 2.8 million deaths per year are attributable to sepsis(3).

**Pathophysiology**

**Sepsis and tissue injury**

**Hypoxia induced injury**

The precise mechanisms of sepsis induced cell injury and organ dysfunction are not fully understood and continue to be an active area of scientific investigation. Tissue ischemia occurs because of either systemic or local mismatch between oxygen delivery and tissue demand.It is interesting that even after aggressive resuscitation of the septic patient, a normal or high cardiac output is typically achieved, yet tissue perfusion can remain markedly impaired. Tissue hypoxia can persist despite achievement of normal or supranormal global oxygen delivery. Clinically, this may manifest with persistent acidosis, mottled skin, or progressive multiorgan failure. **This indicates that sepsis-induced hypoxia is mainly a disorder of the microcirculation.** Therefore, limiting goal‐directed resuscitation solely to macrocirculatory perfusion indices alone may not be sufficient to optimize blood flow to tissues in many patients. The microcirculation (blood vessels< 100μm in diameter) is the principal site of oxygenexchange between blood and underlying tissues, and there is profound disruption of microcirculatory homeostasis in sepsis. Novel therapeutic strategies need to go beyond global hemodynamic optimization aiming to augment microvessel blood flow in patients with sepsis(4).

**Tissue hypoxia, caused by microvascular dysfunction, mitochondrial dysfunction and apoptosis are all thought to be important mediators of sepsis induced multiple organ dysfunction** (3).

Importantly, the degree of microvascular disturbance and its persistence is associated with worse outcomes in septic patients. Small vessel perfusion has been found to be poor in septic patients dying from shock or due to persistent multiple organ failure as compared to survivors (5).

Hypoxia may also enhance tissue injury by increasing viral load in cells, a mechanism which has not been appreciated. Virus and hypoxia share common cellular kinase signaling pathways which on one hand facilitate viral entry and replication and on the other hand promote cellular apoptosis (6). This recognition probably will change the concept of sepsis treatment. Thus, treatments, which target hypoxia induced apoptosis, may be also effective in reducing viral load. Emerging evidence supports this notion (7, 8) (Figure 1).

**Viral induced injury**

Intracellular kinases link external stimuli with downstream effectors through phosphorylation of proteins and /or lipids. Upon stress, pro- apoptotic signaling such as p38MAPK is activated and results in cell apoptosis. Hypoxia induces sustained activation of p38MAPK and tissue injury while interventions that suppress p38MAPK activity increase cell survival (9, 10). Viruses, such as influenza A (IAV), can also replicate via p38MAPK activation, the same pathway which leads to cellular apoptosis (6, 11). **Interestingly, inhibition of p38MAPK is shown to diminish viral replication, vRNP export and apoptosis.** Here, it should be noted that anti-malarial agent hydroxychloroquine attenuates tissue injury by inhibiting p38MAPK activity induced by TNF–a (7). TLR4 mediated viral activation of p38MAPK seems to be important for viral entry and replication (11). Influenza virus, likehypoxia, induces perturbations of the intracellular redox balance resulting in increased production of reactive oxygen species (ROS) which can also activate p38MAPK. Additionally, NADPH oxidase 4 (NOX 4) regulated p38MAPK activation can result in increased ROS production. **Interestingly, female mice exhibited reduced clinical symptoms and viral titers due to gender dependent NOX4 expression (12)**. In contrast higher IAV replication in male mice correlated with higher expression of NOX4 and p38MAPK activity(12). This experimental evidence offers a plausible explanation for the early reports on COVID19 showing that 67% of infected patients were men (Figure 1).

This new evidence on the pathophysiology of sepsis and viral infections probably indicate that pharmaceutical interventions which target kinase signaling pathways mediating both hypoxia and virus-induced cell injury may be novel and effective treatments for sepsis/septic shock. This is an urgent unmet need since the current available treatments are now realized that **can aggravate tissue injury and/or increase viral load.** This probably explains the increased mortality reported in ICU patients infected with COVID 19 (Figure 1).

*Figure 1. Virus and hypoxia-induced tissue injury is mediated via activation of p38 MAPK. Pantos et al. 2020 (13).*

**Drug induced injury**

According to surviving sepsis campaign guidelines inotropes and vasoactives are recommended as therapeutic modalities in the treatment of sepsis (14). However, there is now emerging evidence that certain agents increase mortality of patients with septic shock. In a series of 417 patients with septic shock, the use of epinephrine and dobutamine was associated with significantly higher in-hospital mortality (15). The effects of epinephrine and dobutamine were time and dose dependent. The use of milrinone was not associated with enhanced mortality. All treatments increased the incidence of atrial fibrillation. None of the treatments was associated with more ICU-free days (15) (Figure 2A).

Similar data have been reported in a recent meta analysis: the use of epinephrine for the management of cardiogenic shock was associated with a 3-fold increase in deaths (16).

The potential mechanisms of the unfavorable effects of certain agents, which are commonly used to support haemodynamics in ICU, are not well understood. However, there is experimental evidence that certain vasoactive and inotropes exert their action by enhancing **p38MAPK activation and thus, aggravate tissue injury and viral load.** Experimental evidence (Department of Pharmacology, Medical School of Athens), indicates that sympathomimetic agents can exert detrimental effects on the ischemic myocardium via p38MAPK in models of myocardial ischemia–reperfusion (17). Thus, **phenylephrine a**dministration (an a1 adrenergic agonist) at reperfusion was found to **aggravate tissue injury by increasing p38MAPK activity**(17) (Figure 2B). This is in accordance to reported clinical data showing that epinephrine use was associated with increased mortality particularly in patients with severe ischemia and high lactate levels due to septic shock (15).

Along with this line, in experimental model of viral myocarditis, **Bosentan,** an endothelin-1 receptor (ET1R) antagonist, used to support haemodynamics, although improved cardiac function, **enhanced viral load and myocarditis severity due to p38 MAPK activation** (18). In contrast, administration of SB203580, an inhibitor of p38 MAPK, **attenuated viral replication in the heart, myocardial damage and preserved cardiac function**(18).

Evidence also suggests that **corticosteroid use** is harmful in critically ill patients with viral infections. Corticosteroids suppress inflammation, but inhibit immune responses and pathogen clearance (19). A systematic review and meta-analysis in 6548 patients with severe influenza infection found that corticosteroid use increased mortality by 75% and length of stay in ICU (19).

***Figure 2.*** *A. Hazard Ratio for mortality among patients who received inotropes and those who did not (15). B. The molecular basis of inotropic and vasoactive agents induced injury under hypoxic/reoxygenation conditions. Apoptosis detection by fluorescent probe optical imaging (red signal corresponds to pro-apoptotic caspase 3 activation). Ischemia-reperfusion results in apoptosis despite restoration of blood flow. T3 at reperfusion results in reduced apoptosis, while phenylephrine (anα1-adrenergic agonist often used in clinical practice to support hemodynamics in ischemic settings) aggravates tissue injury. This response is attributed to differential activation of the pro-apoptotic p38 MAPK (10).*

*PE= phenylephrine, T3=triodothyronine, CNT=untreated hearts, R=reperfusion*

**Sepsis and neurohormonal response**

Sepsis imposes a severe stress to the body and leads to an altered neurohormonal response with important physiological consequences. Parallel to the ACTH-Cortisol axis, the body exhibits stress-related changes in thyroid hormone metabolism which result in low T3 levels in serum with normal T4 levels in less serious conditions and both low T3 and T4 serum levels in more severe cases. This response is known as the Non-thyroidal Illness Syndrome (NTIS) and seems to be an important determinant of survival in septic patients (20, 21).

**NTIS and outcome in sepsis**

NTIS affects 60-70% of critically ill patients and is found nearly in 67% of sepsis patients. A meta-analysis of recent studies in patients with sepsis has clearly pointed out that circulating T3 was lower in non survivors versus survivors (20). Furthermore, mortality in septic patients was found to be 13,4% in patients without thyroid hormone abnormalities, 50% in patients with low circulating T3 and rises up to 69,1% in patients with both circulating low T3 and T4 (21). Furthermore, low T3 levels were associated with the risk of adverse cardiovascular events in adult patients with viral myocarditis (22) (Figure 3).

These observations are in line with other acute pathological conditions, such as myocardial infarction, stroke etc., indicating that changes in thyroid hormone metabolism in acute illness are not indicators of disease severity but are implicated in the pathophysiology of the stress response.

Upon stress, an interaction between thyroid hormone signaling and the adrenergic system and immune system occurs and results in suppression of thyroid hormone(23, 24). Changes occur in the conversion of T4 to T3, in the de-gradation of thyroid hormone in tissue and in thyroid hormone nuclear receptors. Furthermore, the hypothalamic-hypophysis-thyroid axis is deregulated (23, 24). This response is of physiological importance. TH regulates several pathways involved in cell differentiation, growth, apoptosis, metabolism and mitochondrial biogenesis(25). Furthermore, contractile proteins such as myosin and calcium handling proteins are thyroid hormone responsive. There is now emerging evidence that TH treatment limits tissue injury and results in organ repair(25). A phase II clinical trial, ThyRepair, is underway investigating the TH healing effects in patients with acute myocardial infarction.

***Figure 3.*** *Kaplan-Meier survival curves in critically ill patients with sepsis (21) . NTIS = Non-Thyroidal Illness Syndrome, Group A (NTIS with low T3 only), Group B (NTIS with low T3 and T4)*

**TH and hypoxia/re-oxygenation tissue injury**

Studies performed for the first time in the Department of Pharmacology, Medical School of Athens, investigated the potential role of TH in tissue injury in models of myocardial ischemia and reperfusion.

**Thyroid hormone effects are not similar on injured and healthy myocardium**

It is now recognized that TH action on the heart depends on its administration on injured or healthy myocardium (26). Ischemic injury has been found to result in hypothyroid-like changes at the tissue level (27). This explains why TH effects are evident at higher doses in ischemic settings.

Thus, in experimental models of ischemia-reperfusion (I/R) using isolated rat heart preparations, T3 administration at reperfusion (at a high dose which had no effect on non ischemic myocardium) resulted in enhanced post-ischemic recovery of function and less myocardial injury as indicated by apoptosis and tissue necrosis markers (10, 26).

In this experimental setting, T4 was shown not to have this protective effect. **Τ3 was found to mediate its protective effect on myocardial injury via the inhibition of p38MAPK activation (10, 26)** (Figure 2B).

These findings resulted in the design of a phase II clinical trial which investigates the effect of high dose T3 in patients with large myocardial infarction (ThyRepair).

Similarly, TH induced protection against injury has been demonstrated in brain, lung, kidney and liver (23, 25). Here, it should be emphasized that **TH increases alveolar fluid clearance in normal and hyperoxia-injured lungs**(28). This is of particular therapeutic importance since ARDS is common in COVID-19 infection.

**Analogies between sepsis and myocardial infarction**

Acute myocardial infarction (AMI) is initiated by occlusion of a large epicardial coronary artery due to atherosclerotic disease. Prompt restoration of blood flow in the occluded epicardial coronary artery by primary percutaneous coronary intervention (PPCI), after an acute ST-segment–elevation myocardial infarction (STEMI), is currently the most effective therapy Despite successful PPCI on large vessels, blood flow may not be restored in all myocardium due to obstruction at the level of small vessels, a phenomenon known as microvascular obstruction (MVO).The injury by myocardial ischemia and reperfusion to the coronary microcirculation is now considered the new frontier in cardioprotection (29, 30). Current evidence suggests that the development of microvascular obstruction (MVO) is associated with adverse left ventricular remodeling, heart failure and worse clinical outcomes (31). In a meta-analysis of 1025 STEMI patients reperfused by PPCI, MVO was associated with the occurrence of a composite of cardiac death, congestive heart failure, and myocardial reinfarction with a hazard ratio of 3.74, whereas Myocardial Infarction (MI) size was not. In a more recent meta-analysis using individual patient data from seven randomized primary PCI trials (n = 1688 patients), **late persistent MVO** assessed after reperfusion by CMR increased hazard ratio for the 1-year composite endpoint of all-cause mortality or heart failure hospitalization (32). This effect was independent from infarct size, age, gender, smoking status and the existence of hypertension, hyperlipidemia and diabetes.

**Microvascular failure is frequent in patients with sepsis and** appears to be a major perturbation with prognostic significance. Severe derangements of microcirculatory flow, including the severity of initial derangements in the early resuscitation phase of therapy as well as the persistence of microcirculatory derangements over time, have been associated with lower survival. A lack of improvement in microcirculatory flow indices early in the ICU course has been associated with multi-organ failure. Thus, microvascular dysfunctionis a triggering event in the development of sepsis-induced multi-organ failure, which is known to be a critical determinant of sepsis mortality (4). Therapeutic approaches to counteract microcirculatory failure could represent a novel strategy to help optimize tissue perfusion in sepsis resuscitation. This remains an unmet need. **According to aforementioned evidence, TH may improve microvascular dysfunction (33, 34).**

***Figure 4****. Persistent Microcirculation dysfunction despite optimal blood flow results to worse outcome both in patients with myocardial infarction and sepsis*

*A. Kaplan-Meier curves showing the time-tofirst event for the primary composite endpoint (MACE) during follow-up according to the cut-off value of late microvascular obstruction (MVO) extent (> 0.385 g). MACE, major adverse cardiovascular events*

*B.Box plot demonstrating the time course of small vessel perfusion in survivors, patients dying in shock, and patients dying after resolution of shock due to persistent multiple organ failure (MOF). The evolution was significantly different between survivors and patients dying in shock or dying after the resolution of shock due to persistent MOF (*analysis of variance, P< 0.05). Small vessel perfusion increased only in survivors (P< 0.05).*

**TH and viral load**

As was previously shown, p38MAPK serves as a common pathway of hypoxia/reoxygenation and virus-induced tissue injury. Thus, it is likely that **TH not only prevents cell injury but also affects viral load via p38MAPK.**

Indeed, it is recently shown that TH can regulate virus gene expression, such as herpes simplex virus (HSV) and modulate latency / reactivation of this virus (35-37). Thus, in a model of herpes viral infectivity in rat, viral load of the hyperthyroid animals was shown to be significantly lower than that of euthyroid (36). Similarly, in vitro presence of supra physiologic levels of thyroxine in the culture media in Vero cell culture decreased viral infectivity. Furthermore, hypothyroid animals showed a significant increase in spleen viral load as compared to that of their euthyroid counterparts(36).In line with this evidence, TH imbalance may affect varicella zoster virus reactivation at different incidence rates in different races and age groups (38) (Figure 4).


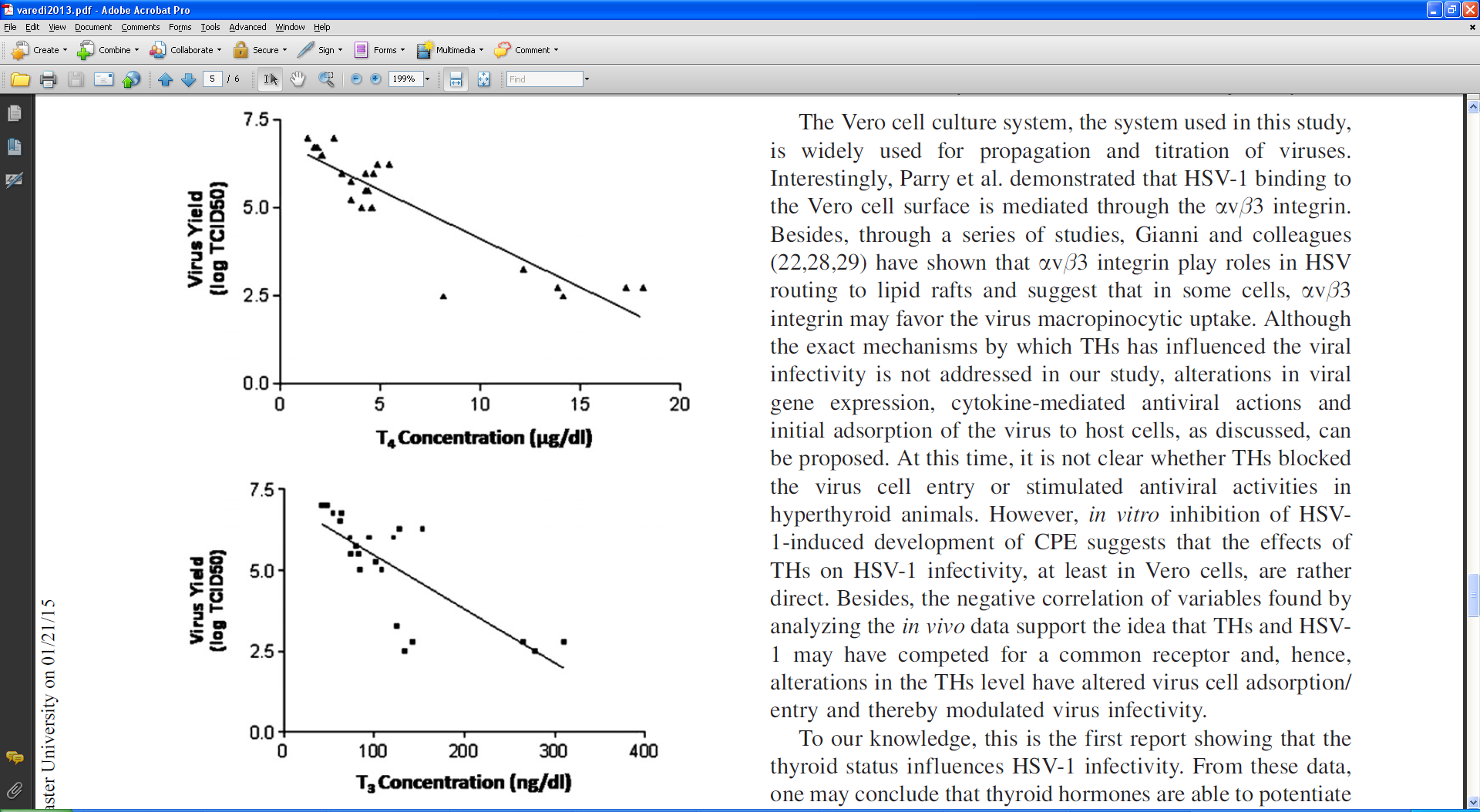


***Figure 5.****Correlation of serum levels of THs with virus yield (TCID50) of animals’ spleen extract. Regression analysis revealed negative relationships between the variables and statistical difference from 0.0 for both hormones.*

**TH and immunomodulation**

TH is critical for immune system function. Even differences within normal range of T3 and T4 levels are associated with changes in the markers of inflammation and immunity. Thus, in a study in healthy individuals, higher ratio of T3/T4 is shown to be associated with increased monocyte phagocytic activity, higher IL-2 receptor density on CD3+ T-lymphocytes and higher absolute counts of NKT cells (39). Furthermore, thyroid hormone treatment modulates T-helper (TH)1/TH2 lymphocyte responses and thereby amplifies host defenses against viral infections (37). Here, it should be noted that T3/T4 is reduced with age (40, 41) and this may account for the susceptibility of the aging group to infectious disease. Indeed, it is shown that administration of TH in aged animals can restore the immune profile to that of the younger counterparts (42).

Furthermore, the potential of thyroid hormone as immunostimulant has been recently shown in patients with stage-I differentiated thyroid carcinoma receiving thyroxine suppressive treatment (43).

TH immunomodulatory effects are of therapeutic importance. Treatments aiming at pan suppression of immune system have failed, as shown by the Myocarditis Treatment Trial (44).

**Aim of the study**

Based on the above evidence, it seems that thyroid hormone is critical in the response to body injury and is now considered as potential pharmaceutical intervention to limit acute tissue injury. TH (via its regulation of stress induced p38MAPK activation) exerts antiapoptotic action and protects tissue from injury, with additional favorable effects on immune system and on viral load in infected tissue. This may be a novel and more effective treatment for critically ill viral infected patients.

ThyRepair is the first study which is underway and investigates the safety and efficacy of high dose T3 treatment in patients with acute myocardial infarction undergoing primary angioplasty. The preliminary reports show that this treatment is safe and the efficacy on tissue repair is promising. This therapeutic modality could also be tested in the acute setting of sepsis since analogies between acute myocardial infarction and sepsis exist.

The safety and efficacy of T3 on heamodynamics in sepsis has been previously demonstrated in a small trial. The present study is phase II, parallel, prospective, randomized, double-blind, placebo controlled trialwhich aims to investigate the potential effect of T3 intravenous use in the recovery of critically ill patients admitted in ICU due to COVID19 infection.

***Figure 5.*** *The potential therapeutic profile of T3 in the treatment of patients with COVID-19 sepsis*

**3. OBJECTIVES**

The ***primary objective*** of the study is to determine whether the administration of intravenous T3 in ICU patients *diagnosed with pulmonary infection due to COVID-19* facilitates weaning from cardiorespiratory support compared to placebo.

**Assessment of weaning from cardiorespiratory support**

- Successful weaning is defined as no requirement for ventilatory support after extubation (mechanical support) or support from ECMO for 48 hours. The primary objective will be measured as percentage of patients successfully weaned after 30 days of follow-up. The percentage of patients weaned at 20 days post-intubation will be a relevant secondary endpoint.

The ***secondary objectives*** of the study are:

1. **Assessment of hemodynamic status**

Hemodynamic status will be assessed by continuous blood pressure measurements (systolic, diastolic and mean BP) and the use of inotropic and vasoactive drugs.

1. **Assessment of pulmonary function**

Pulmonary function will be assessed by arterial measurement of blood gases, Horowitz-Index (pO2/FiO2) and lactate.

1. **Assessment of hepatic and renal function**

Hepatic and renal function will be assessed by laboratory measurements in blood and urine volume

1. **Assessment of cardiac function**

Echocardiographic assessment of systolic and diastolic function will be performed. Measurements of cardiac troponin-I will be used to assess myocardial injury

1. **Assessment of the course of** *COVID-19* **infection**

COVID-19infection will be assessed by inflammatory indices and their course (daily changes and absolute values) in blood (IL6, Procalcitonin, D-Dimers, Ferritin and CRP), temperature monitoring and time needed for the patient to become negative in COVID-19.

1. **Assessment of clinical outcome and safety**

Major events (*death, cardiac Arrest, electromechanical dissociation, pulmonary embolism, new myocardial infarction, stroke, pulmonary edema, cardiogenic shock and hypotension, septic shock, pulmonary embolism, serious bleeding*) and minor (*myocarditis, Venous Thromboembolism, left Ventricular mural thrombus, renal failure, hepatic failure, stress ulcers, minor bleeding,* [*paroxysmal supraventricular tachycardia*](http://emedicine.medscape.com/article/156670-overview)*and*[*atrial fibrillation*](http://emedicine.medscape.com/article/151066-overview)*, rythm disturbances*) will be recorded during the follow up period.

**4. INCLUSION AND EXCLUSION CRITERIA**

In this prospective study all patients diagnosed with COVID19 pulmonary infection receiving endotracheal [mechanical ventilation](https://www.sciencedirect.com/topics/medicine-and-dentistry/artificial-respiration) or ECMO will be considered for enrolment in the study. The study protocol will be approved by the institutional review board and all included patients or legal representatives will provide informed consent to participate in the study.Experimental and clinical data show that maximum efficacy of T3 is achieved when the drug is administered early under hypoxia or hypoxia/reoxygenation conditions where production free oxygen radicals is increased. Thus, the study aims to provide rapid initiation of treatment with T3 within 1 hour of intubation.

In accordance with Good Clinical Practice, all patients will be treated according to the existing guidelines for management of ARDS in patients in ICU requiring [mechanical ventilation](https://www.sciencedirect.com/topics/medicine-and-dentistry/artificial-respiration) or ECMO and patients with septic shock (14, 46, 47).

"Recent guidelines on mechanical ventilation in ARDS provide evidence-based recommendations related to 6 interventions, including low tidal volume and inspiratory pressure ventilation, prone positioning, high-frequency oscillatory ventilation, higher vs lower positive end-expiratory pressure, lung recruitment maneuvers, and extracorporeal membrane oxygenation" (46)

In order to avoid masking the favorable action of T3, we exclude patients that have already received corticosteroids or sympathomimetics which increase viral load and their administration has been associated with increased mortality in sepsis. However, if corticosteroids or sympathomimetics are considered necessary during ARDS treatment of ICU stay, they can be added after administration of the investigational drug has started.

**4.1 MAIN INCLUSION CRITERIA**

- *Patients diagnosed with pulmonary infection and/or ARDS (COVID-19) due to SARS-Cov2viral infection, admitted in ICU and require mechanical ventilation or ECMO*
- *Male and female with Age>18 years old*
- Consent to use an effective contraceptive method beginning from patient enrolment and during participation in the trial, if potential fertile women are enrolled
- *Signed informed consent*

Potential fertile women enrolled in the study will undergo a pregnancy test that will exclude any possibility of present pregnancy. As potential fertile woman is considered every woman who has a menstrual cycle and has not been subjected in surgical sterilization or is not in a post-menopausal condition. In the case of pregnancy, the patient will immediately and automatically be excluded from enrolment and all necessary assistance will be guaranteed for the mother and the child and the pregnancy will be followed until the delivery.

**4.2 MAIN EXCLUSION CRITERIA**

- *Pregnant or breast-feeding women*
- *Known hypersensitivity in the active substance or any of the excipients*
- *Severe systemic disease (e.g. cancer) before infection accompanied by reduced life expectancy <6 months*
- *Participation in another trial of an investigational drug or device*
- *Corticosteroid Use before initiation of treatment*
- *Sympathomimetic Use before initiation of treatment (epinephrine, norepinephrine, dobutamine, dopamine, phenylephrine)*

**5. Data Collection Procedures**

**5.1 Weaning from cardiorespiratory support**

Weaning from ventilatory support plus extubation (in non-tracheostomised patients) will be attempted, when clinically deemed feasible by the treating intensivist. Level of consciousness, muscle strength, ability to cough and protect airways, renal function and fluid balance will be assessed during an attempt to wean. Successful weaning will be defined as no requirement for ventilatory support for 48 hours after extubation, or (in tracheostomised patients) discontinuation of ventilatory support or no need for ECMO support for 48 hours.

**5.2 Echocardiography**

Echocardiographic assessment will be performed as part of the standard clinical care whilst the patients are still in hospital.

Images will be obtained using a 3.5 MHz transducer, at a depth of 12 to 20 cm in parasternal and apical views. Three cardiac cycles of the apical 4-, 3-, and 2-chamber views will be captured in 2D. Left ventricular volumes will be measured and the biplane Simpson’s rule will be applied.

Echocardiographic assessment of diastolic function (mitral E wave velocity/mitral annular velocity (E/E') ratio) and pulmonary congestion (PASP and IVC measurements) will be performed.

All echocardiographic data will be stored digitally and kept center for a centralized core lab reading. Two blinded expert operators will centrally assess data on a specific workstation.

**5.3 Measurement of tissue hypoxia and myocardial injury via troponin release**

Blood samples will be collected, centrifuged under standard conditions and cardiac troponin I will be determined in serum. Lactate concentration in arterial blood will be measured as an index of tissue hypoxia.

**5.4 Measurement of thyroid hormone levels**

Serum total T3, total T4 and TSH will be determined by standard immunoassays at different timepoints: baseline and at 24h, 48h, 72h, and every 3 days till termination of drug administration or end of follow-up. All samples for thyroid hormone measurements will be collected and analyzed at the end in order not to break the blindness of the trial. The coefficients of variation for each assay will be less than 5 percent. The cost of thyroid hormone measurements will be covered by the sponsor.

## 5.5 Recording and evaluation of clinical safety criteria

The following safety clinical criteria will be evaluated:

- Death
- Sudden cardiac death
- Cardiac Arrest
- Electromechanical dissociation
- Pulmonary Embolism
- Venous Thromboembolism
- Myocarditis
- Left Ventricular mural thrombus and/or embolism
- Congestive heart failure
- Cardiogenic shock and hypotension
- Septic shock
- New myocardial infarction
- Stroke
- Renal Failure
- Hepatic Failure
- Stress Ulcers
- Bleeding
- Thrombocytopenia
- Disseminated intravascular coagulation
- Ventricular fibrillation or Sustained ventricular tachycardia
- Rythm disturbances and serious bradycardia that require a pacemaker implantation
- [Paroxysmal supraventricular tachycardia](http://emedicine.medscape.com/article/156670-overview), [atrial flutter](http://emedicine.medscape.com/article/151210-overview), and [atrial fibrillation](http://emedicine.medscape.com/article/151066-overview)
- Vital signs (heart rate, blood pressure)

## 5.6 Recording and evaluation of adverse events

Individual adverse events (AE) will be evaluated by the investigators and they will be reported to the sponsor and Chief Investigator. This includes the evaluation of seriousness, causality and expectedness and any relationship between the investigational medicinal product(s) and/or concomitant therapy and the adverse event. Adverse events will be recorded during hospital stay and till the end of 30 days follow-up. A 24h telephone line will be available for all patients in order to communicate with a study investigator and report any adverse event during the follow up period if necessary.

## 5.7 Recording and evaluation of laboratory and instrumental safety criteria

A number of laboratory measurements will be performed in blood samples in order to evaluate disturbances in blood cells, renal dysfunction, hepatic dysfunction and dysfunction in glucose homeostasis.

The following parameters will be measured Haemoglobin, white blood cells, platelets, urea, creatinine, Glucose, CPK, LDH, AST, ALT, γ-GT, Total and Direct bilirubin, Albumin and total protein, INR, fibrinogen, d-dimer, Electrolytes (Na, K, Ca, phosphate, Mg), Troponin I, BNP, urine volume.

12-leads ECG will be performed every day during ICU stay and every 2 days during hospital stay to assess basal heart rate and cardiac rhythm.

Pulmonary function will be assessed by regular measurements of blood gases (pO2, pCO2, blood pH,lactate).

The course of *COVID-19* infection will be assessed by regular temperature monitoring, inflammatory indices (white blood cells, CRP, IL6, Procalcitonin, Ferritin, D-Dimers) and positivity for active replication of *COVID-19.*

**6 ENROLLMENT OF PATIENTS**

**Number of patients planned to be enrolled**

The primary end-point assessed in the present study will be the percentage of patients successfully weaned after 30 days of follow-up. According to reports that have been published in critically ill patients with COVID19 infection the percentage of patients successfully weaned after mechanical ventilation is low and approximately 15% (1,2). We assume that T3 treatment can increase successful weaning to 50% of patients which is a significant clinical improvement and is close to the mean percentage of successfully weaned sepsis patients (14). With these assumptions the sample size of 60 patients (that indicates 30 subjects for each group) will have **84**% power to detect the estimated difference between the two study groups. The criterion for significance (alpha) has been set at 0.05 and the test is 2-tailed.

***Figure 6.****Graph showingSample size estimation*

This study will start in ICU center of “ATTICO” University Hospital. We aim to include two more clinical sites in the trial one from Greece and one from Germany.

**7 WITHDRAWAL OF PATIENTS AND DEVIATION FROM THE PROTOCOL**

**Specification on criteria for withdrawal of patients: Patient and Investigator criteria**

Clinical follow-up will be performed for all patients randomized in the study even when they are withdrawn for any reason. If for any patient either the study treatment or observations were discontinued the reason will be recorded and documented in the CRF. Withdrawn subjects will be replaced and after the end of the study the number and reasons of withdrawn subjects will be reported both in treatment and placebo group.

Reason that a patient may discontinue participation in this clinical study are considered to constitute one of the following:

- Subject or relative withdrew informed consent
- Unsatisfactory adherence to study protocol or protocol violation (including drug dosage administered, and visit attendance)
- Appearance of clinical status that render the patient unsuitable to continue the study
- Adverse effects related to drug/placebo administration
- Major (cardiac and non-cardiac death, cardiac arrest, reinfarction) and minor (potentially life-threatening arrhythmias) adverse events during the period of drug/placebo administration
- Necessity of therapy with drugs interfering with T3 such as amiodarone or anti-thyroid drugs.

Analysis of data collected programmed upon completion of follow-up of the first 30 patients to eventually discontinue the study in the case of excess adverse events or mortality. In the case of occurrence of a major event, drug/placebo administration will be promptly stopped.

The subject may withdraw from the study at any time without explanation, without losing the right to future medical care. The participation of the subject may, at any moment, be terminated by the investigator, if considered appropriate.

**Any deviation from the protocol (to be classified as Major and Minor) will be accepted only in case of emergency and/or after agreement with the Sponsor.**

**8. RANDOMISATION AND BLINDING PROCEDURES**

**Specification on methods and procedures of randomisation**

This is going to be a double blind study. Thus, trial investigators, medical and nursing staff as well as recruited patients will be blinded to allocation.The representative of the sponsor and the principal investigator will get a copy of randomization codes. The information of the randomization codes will then be locked in the database until the time at which an interim analysis or final analysis is performed.

In order to maintain blindness identical packs with identically appearing contents will be used.

When a patient meets ALL inclusion criteria and NONE of the exclusion criteria, he/she can be enrolled in the study. An allocation sequence to one of the above groups will be prepared by the Sponsor of the study. All patients eligible for the study will be randomized to one of the two groups.

A 1:1 treatment allocation will be adopted. Randomization number – and thus the type of formulation – is assigned to each participating patient randomly, depending on the time of arrival to the site and enrollment and ONLY after written informed consent.Randomization data will be kept strictly confidential, only accessible to authorized persons. The blind experiment can cease only due to adverse side events or if essential for the safety of patients. Trial investigators, medical and nursing staff as well as recruited patients will be blinded to allocation.

**Specification on procedures for blindness**

In order to maintain blindness identical packs with identically appearing contents will be used for both placebo and drug administration.

The investigator site personnel involved in the monitoring or conducting of the trial will be blinded to the trial drug codes. Trial drug codes will not be available to the above personnel until after the completion of the trial and final data review, except in the case of an emergency.

Specification on situation, procedures and responsibilities for the unblinding

Randomization data will be kept strictly confidential, only accessible to authorized persons, until the expiry date. Emergency unblinding can occur only due to adverse side events or if essential for the safety of patients. Unblinding is only done when it is deemed essential for the patients by the subject’s physician, by a clinical investigator or by the Data Monitoring Committee.

The investigator should evaluate serious AEs for causality and expectedness as though the patient was on the active drug. Only cases that are considered serious, unexpected and possibly, probably or definitely related (like a SUSAR) would have to be un-blinded for reporting to the competent authorities.

A request for unblinding a subject could be done by:

(a) a physician who is not directly involved in the management of the specific clinical trial

(b) a clinical investigator who is directly involved in the management of the patient in this trial or

(c) the Data Monitoring Committee (DMC). A DMC will have access to unblinded data and can oversee the assessment of emerging risks such as increase in severity or frequency of expected events.

The procedure for unblinding is described below:

- The clinical investigator or treating physician contacts by phone the holder of the code break envelope/list. The on-call 24h authorized representative of the sponsor is designated as the holder of the code break envelope/list responsible for this action.
- The authorized representative of the sponsor provides the CI or treating physician with the information as requested.
- The study code should only be broken when absolutely necessary for valid medical or safety reasons e.g. in the case of a severe adverse event where it is necessary for the CI or treating physician to know which treatment the patient is receiving before the participant can be treated.
- The CI documents the breaking of the codes as per protocol.
- On receipt of the treatment allocation details, the CI or treating physician deals with the participant’s medical emergency as appropriate.
- If the treating physician is not the CI, the treating physician must inform the CI of the code break and the reasons for the actions taken as soon as possible.
- The CI documents the breaking of the code and the reasons for doing so on the CRF.
- The authorized representative of the sponsor documents the breaking of the code and the reasons for doing so on the code list within the Pharmacy study file.
- Authorized representative of the sponsor notifies the Research and Development Department in writing as soon as possible following the code break detailing the necessity of the code break and copies to the Coordinator.
- The CI notifies the Research Ethics Committee of the protocol deviation and copies the letter to the Research and Development Department.
- Un-blinded SUSARs will be reported to the appropriate regulatory bodies.

In the event of un-blinding the CI must document the action taken and promptly notify the DMC. Notification must include:

- Name and title of person who requested the code break
- Subject number
- Reason for un-blinding
- Date and time of code break
- Name of person authorizing the procedure
- Signature of person performing the un-blinding.

**Breaking the blind at the end of the study**

The un-blinding of participants cannot occur until all participants have completed the final follow-up (also known as Last Patient Last Visit, LPLV).

Un-blinding should not take place until the database has been locked i.e. all data entered, validated and no further changes are expected. Furthermore, the person performing the statistical analysis will remain blinded until after the analysis has been completed.

Procedure for unblinding at the end of the study is described below:

The principal investigator (PI) of the center contacts the DMC. DMC confirms that the study may be un-blinded by email to authorized representative of the sponsor and provides treatment allocation details to the PI as requested.

If necessary, (e.g. for safety reasons) the PI should make every effort to inform all-participants of their individual treatment assignment.

The PI determines the appropriate method of informing participants of the blinded random assignment.


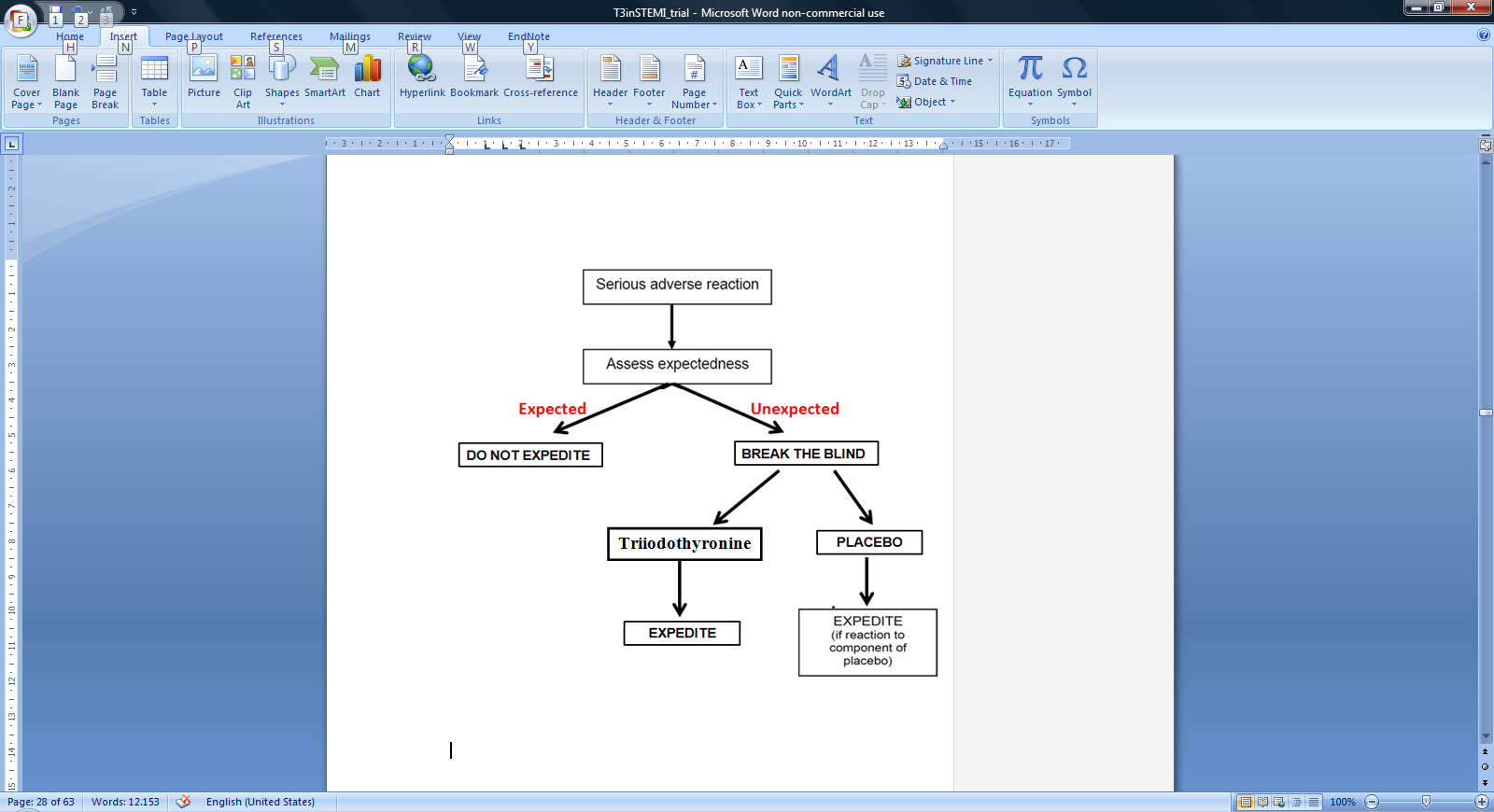


***Figure 7.*** *Schematic plan of serious sdverse drug reactions handling during the trial*

**9. TREATMENT**

### Route of Administration

Intravenous use.

### Maximum duration of treatment of a subject

Each subject will be treated until successful weaning or end of follow up. The maximum treatment duration of a subject may reach 30 days.

### Active comparator products

*Product under investigation:*

T3^®^ Solution for injection 10 μg/ml, each vial contains 150μg of T3 in a total volume of 15ml.

*Placebo:*

Composition identical apart from the active substance.

### Procedures for monitoring subject compliance

Triodothyronine sodium will be administered via intravenous pump and compliance will be monitored continuously during the period of administration from specialized personnel. In addition, measurements of thyroid hormone levels in serum will document both subject compliance and correct practice of the investigators.

##

## Dosage and Safety

## Experimental and clinical data show that maximum efficacy of T3 is achieved when the drug is administered early under hypoxia or hypoxia/reoxygenation conditions where production free oxygen radicals is increased. Thus, the study aims to provide rapid initiation of treatment with T3 within 1 hour of intubation.

## The initial dose for the first 48hours is a high dose of T3 (therapeutic dose) exactly the same with the dose used in ThyRepair trial. Data show that this dose is safe (see below). Administration of T3 beyond 48 hours was not necessary in ThyRepair trial since these patients maintain T3 levels within normal range due to stable hemodynamic status and absence of other stress factors. In ThySupport trial, after the first 48hours, administration of T3 will continue with a maintenance dose which will be reduced by 50%. This is necessary because prolonged mechanical respiratory support and sepsis in these patients inhibit conversion of T4 to T3 and result in sustained reduction in T3 levels.

## Thus, in ThySupport, the dose administered will be 0.8μg/kg i.v. bolus, starting within 60min after respiratory support and will be followed by an infusion of 0.113μg/kg/h i.v. for 48 hours (therapeutic dose). After the first 48h, a maintenance dose will be administered corresponding to 50% of the therapeutic dose (0.057 μg/kg/h i.v.). Drug administration will stop after successful weaning or end of followup (maximum 30 days).

***Figure 8.*** *Schematic of drug administration schedule inThySupport study*

In previous studies in CABG patients it was shown that administration of the dose of T3 proposed here (0.8μg/kg at the end of ischaemia followed by an infusion of 0.113μg/kg/h) resulted in short-term supranormal serum T3 levels without any effects on heart rate or arrhythmias or any other adverse event reported.


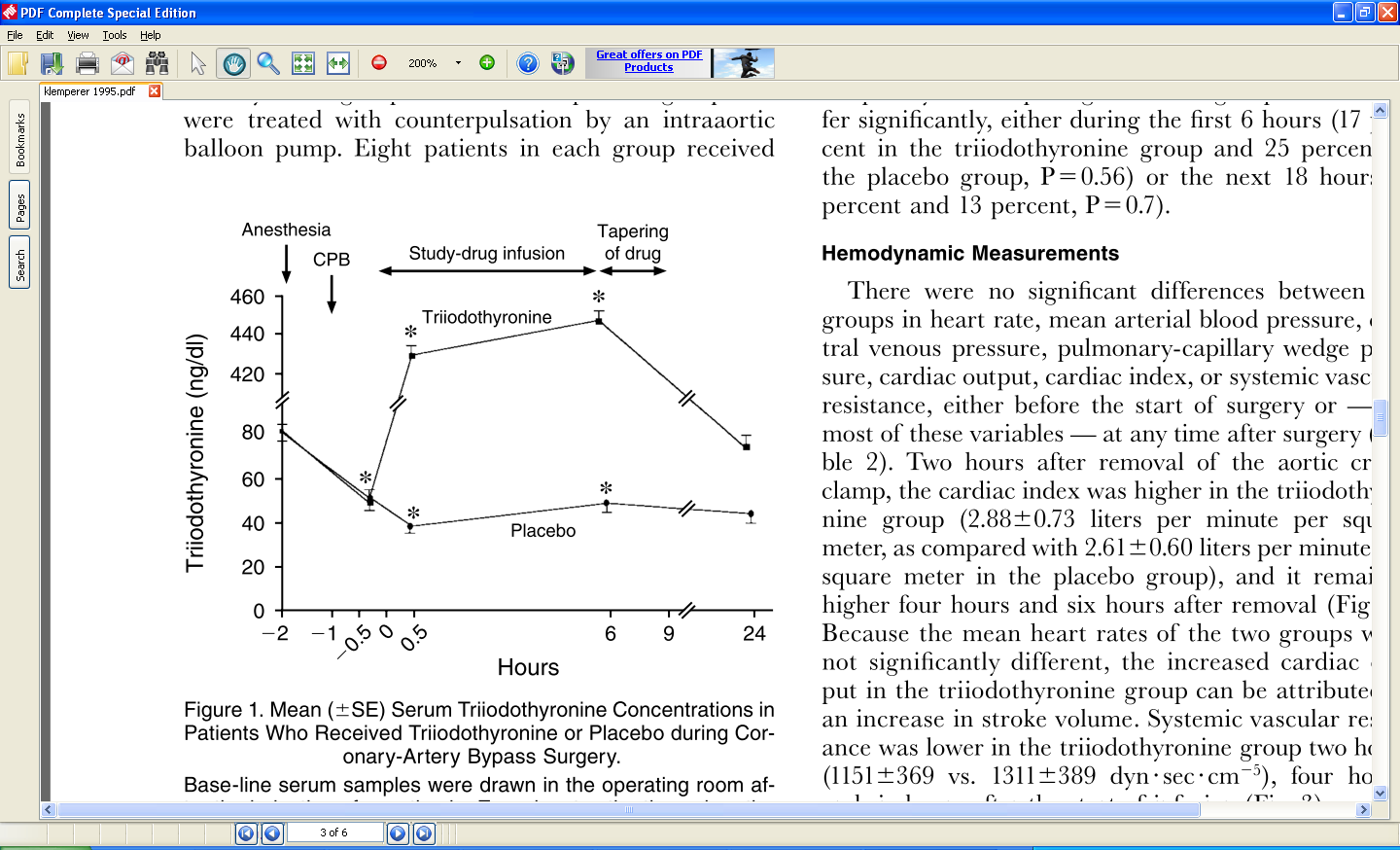


***Figure 9.*** *Pharmacokinetics of T3 in CABG patients*

More importantly, data from the ThyRepair study (EudraCT: 2016-000631-40) conducted in patients with ST-elevation myocardial infarction showed that administration of the drug under investigation at the proposed doses (0.8μg/kg i.v. bolus followed by an infusion of 0.113μg/kg/h i.v. for 48 hours) resulted in significant increase in the levels of T3 above normal that lasted for 72 hours without any significant adverse event.


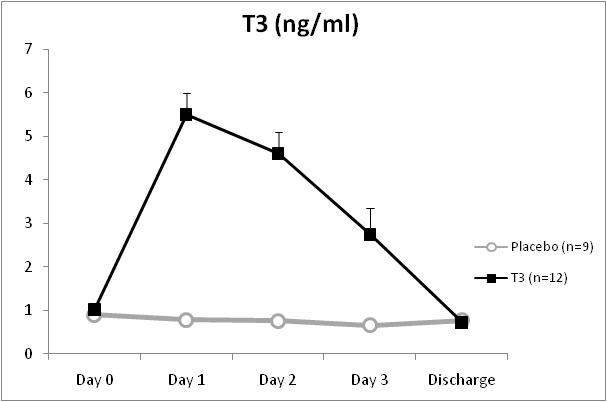


***Figure 10****: Levels of triiodothyronine in serum during hospitalization in patients after acute myocardial infarction treated with placebo and T3*

Administration of high dose T3 resulted in a non-significant increase in heart rate during the first 3 days (Figure 11).

***Figure 11****: Heart rate assessed during hospitalization in STEMI patients treated with placebo and T3*

***Table.*** *Adverse events during hospitalization in Thy-Repair trial (interim analysis).*

| **Adverse Events** | **placebo Group**  **(n=17)** | **T3 Group**  **(n=16)** |  |
| --- | --- | --- | --- |
| Atrial Fibrillation | 7.7% | 19% | *p=0.25* |
| Thrombus in Left Ventricle | 12% | 12.5% | *p=0.94* |
| Pericardial Effusion | 6% | 6.25% | *p=0.9* |
| Minor Hemorrhages | 12% | 19% | *p=0.57* |
| Diuretics Use during first 24 hours | 40% | 50% | *p=0.7* |
| Need for Sympathomimetics administration after PPCI | 30% | 19% | *p=0.45* |
| Transient Fever wave>37.8 | 0% | 31% | *p=0.01* |

## A non-significant trend for increased episodes of atrial fibrillation in patients treated with T3 has been shown in ThyRepair. Here, it should be noted that the use of inotropes such as epinephrine and dobutamine has been recently found to increase the odds of atrial fibrillation by 3.88 and 3.95 in pateints with septic shock.

## The increased temperature that was observed in some patients in ThyRepair study involved a single fever wave up to 38.5 during the second day of treatment that receded after paracetamol administration. This wave was recorded in 1 to 3 patients receiving T3. It didn’t cause any diagnostic issues since other inflammatory indices did not increase.

Thyroid hormones are eliminated 70-80% by the kidneys. In ThyRepair study we enrolled patients with GFR more than 60 ml/min and no problem was recorded after T3 administration. A reduction of GFR<30ml/min could increase T3 levels in blood. However, this increase is blunted by attenuation of the conversion of T4 to T3 which is reported under conditions of acute or chronic renal failure (37). It should be noted that the therapeutic index of T3 is high. In case there is a potential adverse event that could be linked to T3 in a patient with severe renal failure, urgent unblinding of the patient can be performed and the levels of T3 can be determined.

The liver has a secondary role in the elimination of thyroid hormones by 25%. Acute or chronic hepatic injury reduces the conversion of T4 to T3. Thus, in patients with hepatic injury, normal or increased levels of T4 and low levels of T3 have been reported. (Kano T, et al.. Serum thyroid hormone levels in patients with fulminant hepatitis: usefulness of rT3 and the rT3/T3 ratio as prognostic indices (38). Based on the above, administration of T3 in patients with hepatic injury will not result in higher than expected levels of T3 in blood.

Further analysis of preliminary data from ThyRepair show that although infarct size was similar between the placebo and T3 group, an improvement of LV ejection fraction is evident in T3 treated STEMI patients early and late after the index event (Figure 12 and 13).

***Figure 12.*** *CMR analysis shows no difference in infarct size (extent of injury) between placebo and T3 group.*


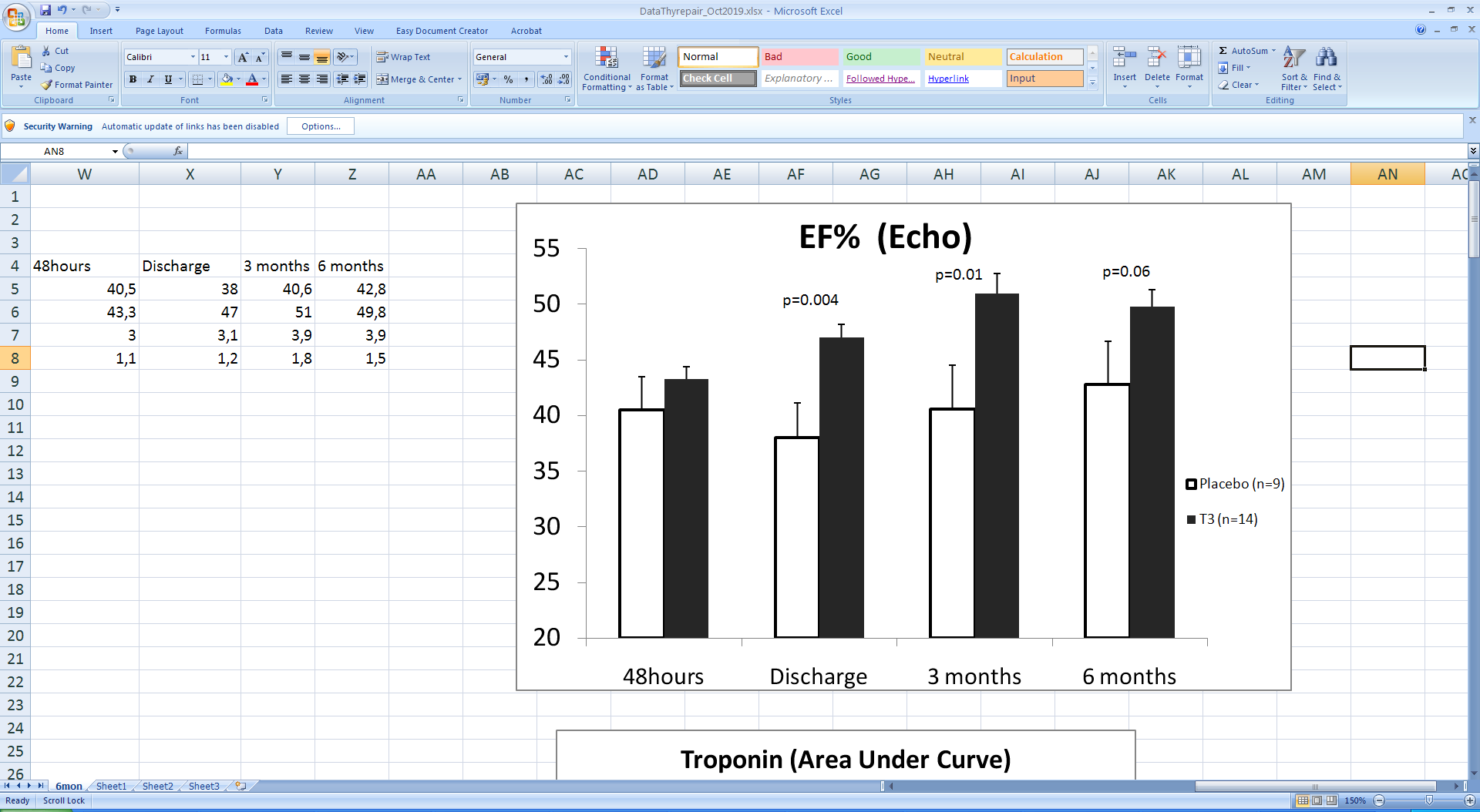


***Figure 13.*** *Left ventricular ejection fraction assessed by echocardiography at different time points in STEMI patients treated with placebo and T3.*

In a preliminary study (48), in 11 patients with severe septic shock that were all treated with dopamine, T3 was administered by continuous intravenous infusion in doses of 100-200μg/24h. In all patients, there was an increase in arterial blood pressure and dopamine dependence was terminated. T3 levels rose during infusion up to concentrations of 4 ng/ml and in some cases patients were treated up to 28 days without presentation of significant adverse events(48).

## Dosage schedules

Dosage schedules are summarized in the following table.

| **Patient weight** | **Bolus administration over 2-3 min** | **Continuous infusion** | **Pump rate**  **(first 48h)** | **Pump rate**  **(from day 3 till end)** |
| --- | --- | --- | --- | --- |
| 66Kg | 5.5 ml (55μg) | 18ml (180μg) in 232 ml NaCl 0.9% | 10.4 ml/h | 5.2 ml/h |
| 70Kg | 5.5 ml (55μg) | 19ml (190μg) in 231 ml NaCl 0.9% | 10.4 ml/h | 5.2 ml/h |
| 74Kg | 6 ml (60μg) | 20ml (200μg) in 230ml NaCl 0.9% | 10.4 ml/h | 5.2 ml/h |
| 77Kg | 6 ml (60μg) | 21ml (210μg) in 229ml NaCl 0.9% | 10.4 ml/h | 5.2 ml/h |
| 81Kg | 6.5 ml (65μg) | 22ml i(220μg) n 228ml NaCl 0.9% | 10.4 ml/h | 5.2 ml/h |
| 85Kg | 7.0ml (70μg) | 23ml (230μg) in 227ml NaCl 0.9% | 10.4 ml/h | 5.2 ml/h |
| 89Kg | 7.0ml (70μg) | 24ml (240μg) in 226ml NaCl 0.9% | 10.4 ml/h | 5.2 ml/h |
| 92Kg | 7.5ml (75μg) | 25ml (250μg) in 225ml NaCl 0.9% | 10.4 ml/h | 5.2 ml/h |
| >95Kg | 7.5ml (75μg) | 26ml (260μg) in 224ml NaCl 0.9% | 10.4 ml/h | 5.2 ml/h |

For example, for a patient of 77Kg of weight, a dose of 6ml (60 μg) will be administered as a bolus intravenously over 2-3 min within 60 min of respiratory support initiation. Then, the patient for the next 24 hours will receive 21ml of the product (total of 210 μg of T3) that will be diluted in NaCl 0.9% and administered with a pump at a steady flow rate of 10.4 ml/h for a total duration of 48 hours. From day 3 till successful weaning or end of follow-up, the patient will receive 50% of this dose, 10.5 ml of the product (total of 105μg of T3)that will be diluted in NaCl 0.9% and administered with a pump at a steady flow rate of 5.2 ml/h.

## Presentation of the drug

The under investigation product *T3^®^Solution for injection 10 μg/ml*is a clear, colorless solution.

*Composition of the Medicinal Product*

| **No** | **Name of ingredient(s)** | **Quantity / 1ml** |
| --- | --- | --- |
|  |  |  |
|  | **Active ingredient:** |  |
| 1. | Liothyronine sodium | 10.0μg |
|  | **Other ingredient (s):** |  |
| 1. | Dextran 70 | 60.0 mg |
| 2. | NaOH 1 N | q.s. pH 10 |
| 3. | Water for Injections | qs 1.0 |

*Proposed shelf life:* 1 month.

*Placebo:* Composition identical apart from the active substance.

## Known Drug reactions

### Oral Anticoagulants: T3 appears to increase catabolism of vitamin K-dependent clotting factors. If oral anticoagulants are also being given, compensatory increases in clotting factor synthesis are impaired. It is likely that a reduction in anticoagulant dosage may be required.

### Heparin: According to the literature, the action of heparin can be affected by T3 adminsitration. In the ThyRepair study, in patients with myocardial infarction receiving antiplatelet and anticoagulant therapies as gold-standard, serious adverse events such as hemorrhages or thrombosis were not observed. In patients in the ThySupport study, we advise to use unfractioned heparin and perform dose adjustment according to aPTT measurements.

### Insulin or Oral Hypoglycemics: Initiating T3 therapy may cause increases in insulin or oral hypoglycemic requirements. The effects seen are poorly understood. Patients receiving insulin or oral hypoglycemics should be closely watched during initiation of T3 therapy.

### Estrogen, Oral Contraceptives: Estrogens tend to increase serum thyroxine-binding globulin (TBG). Therefore, hypothyroid patients who are on thyroid replacement therapy may need to increase their thyroid dose if estrogens or estrogen-containing oral contraceptives are given.

### Tricyclic Antidepressants: Use of thyroid products with imipramine and other tricyclic antidepressants may increase receptor sensitivity and enhance antidepressant activity; transient cardiac arrhythmias have been observed. Thyroid hormone activity may also be enhanced.

### Digitalis: Thyroid preparations may potentiate the toxic effects of digitalis. Thyroid hormonal replacement increases metabolic rate, which requires an increase in digitalis dosage.

### Ketamine: When administered to patients on a thyroid preparation, this parenteral anesthetic may cause hypertension and tachycardia. Its use should be avoided.

### Vasopressors: Thyroid hormones increase the adrenergic effect of catecholamines such as epinephrine and norepinephrine. Therefore, patients receiving T3 may need lower doses of vasopressors.

## In the ThyRepair study, administration of T3 at the same therapeutic doses in patients with STEMI who received at the same time several other drugs (antiplatelets, anti-coagulants, β-blockers, ACE inhibitors,ARBs, aldosterone antagonists, statins, diuretics, insulin etc,) did not show any serious issue of drug reactions between T3 and other therapies.

## Drug storage and supply

The under investigation product and the placebo will be supplied by the Sponsor.

## *Proposed Storage conditions:* Store under 2-8^o^C.

**10.** **FLOW-CHART OF THE STUDY**

**General schedule for Patient and Investigator**

Patients with COVID19 pneumonia on mechanical ventilation or ECMO, n=90

Follow-up for 1 month, n=30

Follow-up for 1 month, n=30

Placebo group, n=30

T3 group, n=30

Randomisation 1:1, n=60

Assessment of eligibility, n=70

Signed inform consent, n=60

**11. STUDY PROCEDURES**

## Informed consent

The investigator must explain to each patient or legal representativethe nature of the study, its purpose, the procedures involved, the expected duration, the potential risks and benefits involved and any inconveniences it may cause.

Each patient or legal representative must be informed that participation in the study is voluntary, consent maybe withdrawn at any time and that withdrawal of consent will not affect subsequent medical treatment or relationship with the treating medical staff.

The Informed Consent Form should be administered by means of a standard statement, written in non-technical language. The patient or legal representative should have time to read and consider the statement before signing and dating it. Patient or legal representative should receive a copy of the signed document. No patient can enter the study before Informed Consent Form has been obtained.

## Screening evaluation

All patients diagnosed with COVID19 pulmonary infection receiving endotracheal [mechanical ventilation](https://www.sciencedirect.com/topics/medicine-and-dentistry/artificial-respiration) or ECMO will be considered for enrolment in the study in the clinical sites. According to our plan, this study will need to screen approximately 90 patients, in order to enrol 60 patients that are eligible according to the inclusion and exclusion criteria and will provide signed informed consent.

Eligible patients with the above criteria will be approached and an informed consent will be asked from patients or relatives. After informed consent has been signed, each investigator has to inform the Chief Investigator within 24 hours about the new patient recruitment. Chief investigator is obliged to evaluate whether all criteria are met.

## Baseline data

All patients will have a full medical history taken and a clinical examination. The following are to be recorded:

- ***Patient profile*** (Height, Weight, gender, Race, Age and date of birth, Address and phone number, significant past medical history, smoking, time from onset of symptoms to admission)
- ***Any Co-morbidities***
- ***Medication at admission in ICU***
- ***Blood pressure and heart rate***
- ***Blood gases*** (pO2, pCO2, blood pH, lactate)
- ***Echocardiographic analysis***
- ***ECG***
- ***Standard Laboratory data:*** *Haemoglobin, White blood cells, platelets, urea, creatinine, glucose, CPK, LDH, AST, ALT, γ-GT, Total and Direct bilirubin, Albumin and total protein, INR, IL6, Procalcitonin, Ferritin, d-dimer,fibrinogen, electrolytes (Na, K, Ca, phosphate, Mg), troponin I, BNP, CRP, erythrocyte sedimentation rate*
- ***24h urine volume***

## Study assessments

***During stay in the ICU the following parameters will be recorded every 24 hours:*** Blood pressure, heart rate, oxygen saturation, temperature, blood gases, urine volume, need for inotropic support, standard medication, laboratory tests, ECG and evaluation of AEs.

**Echocardiographic analysis** will be performed every 48 hours during ICU stay

**Thyroid hormone levels** (Serum total T3, total T4 and TSH) will be determined at baseline and at 24h, 48h, 72h, and every 3 days till termination of drug administration or end of follow-up.

**Successful weaning** will be defined as no requirement for ventilatory support for 48 hours after extubation, or (in tracheostomised patients) discontinuation of ventilatory support or no need for ECMO support for 48 hours. The percentage of successfully weaned patients and the duration of required mechanical respiratory support or ECMO (in days) will be recorded.

**Individual adverse events** (AE) will be evaluated by the investigators and they will be reported to the sponsor. This includes the evaluation of seriousness, causality and expectedness and any relationship between the investigational medicinal product(s) and/or concomitant therapy and the adverse event. Adverse events will be recorded during 30 days follow-up. A 24h telephone line will be available for all patients in order to communicate with a study investigator and report any adverse event in the case of discharge from hospital.

**12. ADVERSE EVENTS**

## Definitions

### Adverse event

Any untoward medical occurrence in a patient or clinical trial subject administered a medicinal product and which does not necessarily have a causal relationship with this treatment [Dir 2001/20/EC Art 2(m)].

An adverse event can therefore be any unfavourable and unintended sign (e.g. an abnormal laboratory finding), symptom, or disease temporally associated with the use of a medicinal product, whether or not considered related to the medicinal product.

### Adverse drug reaction (ADR)

A response to a medicinal product which is noxious and unintended [DIR 2001/83/EC Art 1(11)]1. Response in this context means that a causal relationship between a medicinal product and an adverse event is at least a reasonable possibility (see Annex IV, ICH-E2A Guideline).

Adverse reactions may arise from use of the product within or outside the terms of the marketing authorisation or from occupational exposure [DIR 2001/83/EC Art 101(1)]. Conditions of use outside the marketing authorisation include off-label use, overdose, misuse, abuse and medication errors.

(All AEs judged by either the reporting investigator or the sponsor as having a reasonable causal relationship to a drug).

1. The relationship between an AE and a drug is considered **Certain** when:

The AE occurs in a plausible time relation to the administration of the drug and cannot be explained by a concurrent disease or other drugs or chemicals. The response to withdrawal of the drug (dechallenge) should be clinically plausible. The event must be definitive pharmacologically or phenomenologically, using a satisfactory rechallenge procedure if necessary.

1. The relationship between an AE and a drug is considered **Probable** when:

The AE occurs in a reasonable time relation to the administration of the drug, it is unlikely to be attributed to a concurrent disease or other drugs or chemicals and it follows a clinically reasonable response on withdrawal (dechallenge). Rechallenge information (AE reappearance after drug reintroduction) is not required to fulfill this definition.

1. The relationship between an AE and a drug is considered **Possible** when:

The AE occurs in a reasonable time relation to the administration of the drug, but it could also be explained by a concurrent disease or other drugs or chemicals. Information on drug withdrawal (dechallenge) may be lacking or unclear.

- The relationship between an AE and a drug is considered **Unlikely** when:

a causal relationship cannot be definitively ruled out, but

- other drugs, chemicals or underlying disease provide plausible explanations

and/or

- the temporal relation to the administration of the drug makes a causal relation improbable.

**An AE is considered Not Related** to a drug in case of:

- existence of a clear alternative explanation, and/or

- unreasonable temporal relationship between Drug and Event, and/or

- non plausibility.

**An AE is considered Unassessable** when:

it cannot be judged, because the information is insufficient or contradictory and cannot be supplemented or verified.

**Intensity of an AE/ADR**

- Mild: does not interfere with routine activities; in case of laboratory tests, when there is a mild abnormality.
- Moderate: interferes with the routine activities; in case of laboratory tests, when there is a moderate abnormality.
- Severe: makes impossible to perform routine activities; in case of laboratory tests, when there is a significant abnormality.

An AE/ADR is considered **Unexpected** when its nature, severity or outcome is not consistent with the information included in the **Summary of Product Characteristics** (SPC).

**Unexpected adverse reaction**

An adverse reaction, the nature, severity or outcome of which is not consistent with the summary of product characteristics [DIR 2001/83/EC Art 1(13)]9.

This includes class-related reactions which are mentioned in the summary of product characteristics (SmPC) but which are not specifically described as occurring with this product. For products authorised nationally, the relevant SmPC is that authorised by the competent authority in the Member State to whom the reaction is being reported. For centrally authorised products, the relevant SmPC is the SmPC authorised by the European Commission. During the time period between a CHMP opinion in favour of granting a marketing authorisation and the Commission decision granting the marketing authorisation, the relevant SmPC is the SmPC annexed to the CHMP opinion.

**Serious adverse event or serious adverse reaction**

Any untoward medical occurrence or effect that:

- results in death,
- is life-threatening
- requires hospitalisation or prolongation of existing inpatients’ hospitalisation,
- results in persistent or significant disability or incapacity,
- is a congenital anomaly or birth defect.

Life-threatening in the definition of a serious adverse event or serious adverse reaction refers to an event in which the subject was at risk of death at the time of event; it does not refer to an event which hypothetically might have caused death if it were more severe.

Medical and scientific judgement should be exercised in deciding whether expedited reporting is appropriate in other situations, such as important medical events that may not be immediately life-threatening or result in death or hospitalisation but may jeopardise the patient or may require intervention to prevent one of the other outcomes listed in the definition above. *These should also usually be considered serious.*

Examples of such events are intensive treatment in an emergency room or at home for allergic bronchospasm; blood dyscrasias or convulsions that do not result in hospitalisation; or development of drug dependency or drug abuse.

## Expected adverse drug reactions

All expected adverse drug reactions and all expected serious adverse events are listed below ***otherwise they will be reported as SUSARs***.

There are possible side-effects associated with triiodothyronine that can affect individuals in different ways. Serious side effects are rare and include an allergic reaction, chest pain, shortness of breath and arrhythmias. In the treatment of myxedema, side effects associated with the use of intravenous T3 have included arrhythmias (5% of patients) and tachycardia (3%). Hypotension and myocardial infarction occurred in approximately 1.5% of hypothyroid patients that received T3. Angina and congestive heart failure occurred in approximately fewer than 1% of patients.

Other, less serious side effects consist of:

- insomnia;
- headache;
- leg cramps;
- diarrhea, abdominal cramps
- seizures, tremors, nervousness, or irritability;
- menstrual irregularities.
- phlebitis
- Nausea or vomiting
- fever, sweating, or heat intolerance;
- changes in appetite, or weight loss;
- Hypertension
- Skin flushing
- Hypoglycemia
- Adrenal insufficiency

Sometimes, unexpected adverse drug reactions and unexpected serious adverse events become ‘expected’ during the trial, in which case the protocol should be amended and such events would not need reporting. The Chief Investigator and Data Monitoring Committee, will determine whether any events become ‘expected’ during the course of the trial and apply for approval for a substantial amendment.

## Expected Serious Adverse Events

*Expected serious adverse events in patients with COVID19 infection requiring mechanical ventilation or ECMO are listed below(2, 3, 14):*

- Death
- Sudden cardiac death
- Cardiac Arrest
- Electromechanical dissociation
- Pulmonary Embolism
- Venous Thromboembolism
- Disseminated intravascular coagulation
- Myocarditis
- Left Ventricular mural thrombus and/or embolism
- Congestive heart failure
- Cardiogenic shock and hypotension
- Septic shock
- New myocardial infarction
- Angina
- Stroke
- Renal Failure
- Hepatic Failure
- Stress Ulcers
- Bleeding
- Thrombocytopenia
- Ventricular fibrillation or Sustained ventricular tachycardia
- Rythm disturbances and serious bradycardia that require a pacemaker implantation
- [Paroxysmal supraventricular tachycardia](http://emedicine.medscape.com/article/156670-overview), [atrial flutter](http://emedicine.medscape.com/article/151210-overview), and [atrial fibrillation](http://emedicine.medscape.com/article/151066-overview)

#

# Toxicity – Emergency Procedures

Significant toxicity with acute ingestions of T3 is rare. Serious toxicity is more commonly observed with chronic ingestions of large amounts of T4.

According to the Annual Report of the American Association of Poison Control Centers’ National Poison Data System, in 2008, 13,005 exposures to thyroid hormone preparations were documented; of the total listed, 9,006 were single substance exposures. The breakdown by age for single substance exposures is as follows; 5,026 were associated with children younger than 6 years; 554 were associated with persons aged 6-19 years; and 2,957 were associated with those aged older than 19 years. Overall, 3 major adverse outcomes and no deaths were reported.

**Procedures in case of toxicity**

Place patient on close cardiac monitoring.

Withdraw trial treatment

Episodes of ventricular fibrillation or sustained ventricular tachycardia will be treated with cardioversion.

Peri-operative myocardial infarction and angina will be treated according to Guidelines of European Society of Cardiology

Beta-blockers will be administered to counteract the increase in adrenergic activity and treat serious tachyarrhythmias. Asymptomatic patients should not be treated empirically with beta-blockers. Propranolol is a first choice treatment. Propranolol is noncardioselective beta-blocker, widely available. Controls cardiac and psychomotor manifestations within minutes (dose 0.01-0.1 mg/kg IV, 2-5min; titrate to effect). Esmolol could be used alternatively. Esmolol is a short-acting IV cardioselective beta-adrenergic blocker with no membrane depressant activity. Esmolol is an intravenous agent with half-life of 8 min, which allows for titration to effect and quick discontinuation (dose 50-200 mcg/kg/min IV; titrate to effect).

Symptomatic patients require correction of dehydration and control of hyperthermia.

Antipyretics will be used to treat hyperthermia. Acetaminophen is a first choice agent for fever control; It inhibits action of endogenous pyrogens on heat-regulating centers; reduces fever by a direct action on the hypothalamic heat-regulating centers, which, in turn, increase the dissipation of body heat via sweating and vasodilation (dose 325-650 mg PO q4-6h or 1000 mg tid/qid; not to exceed 4 g/d). Aspirin is contraindicated because it displaces T4 from thyroid-binding globulin (TBG), increasing free T4.

Corticosteroids can be used when adrenal crisis is suspected. Hydrocortisone will be used to treat the potential adrenal insufficiency occurring secondary to the hypermetabolic hyperthyroid state (dose 100 mg IV bolus, followed by continuous infusion of 100 mg 8h for 24-48 h).

### Reporting Duties of the Investigator

The Investigator must report any Serious AE (whether or not thought to be related to the investigational drug) by faxing the CRF-AE recording pages **no later than 24 hours** after knowledge of the event to:

**Sponsor’s representative responsible for safety**:

Georgios-Stefanos Soumelas

Sponsor’s project manager and responsible for product safety

Tel: 2108072512

Tel (24h): 6937486073

Fax: 210 8078907

email: soumelas@uni-pharma.gr

All Serious AEs shall be followed-up until its outcome.

Any information and supporting documentation that become available (copies of laboratory reports, tests, procedures, autopsy evidence of the cause of death, etc.) shall be provided by the Investigator to the responsible for safety sponsor’s representative with additional written reports as soon as possible.

The Investigator must immediately communicate any case of pregnancy occurred during the duration of the study. If the pregnancy results in an abnormal outcome, the case should be managed as a Serious AE and reported **no later than 24 hours** after the knowledge.

The Investigator must also comply with the local applicable obligation(s) on the reporting of ADRs to the local concerned Regulatory Authority/ Ethic Committee.

###

### Reporting Duties of Uni-Pharma

Uni-Pharma shall ensure that all relevant information about any Serious and Unexpected Adverse Events considered at least “Possible related” to triiodothyronine (SUSARs) will be reported to the Competent Authorities and Ethics Committees (following general and local rules and procedures), with these deadlines after the knowledge:

- fatal and life threatening unexpected cases, no later than 7 days;
- other unexpected serious cases, no later than 15 days.

Uni-Pharma shall ensure that all information and supporting documentation that subsequently become available, will be also reported as soon as possible to the Competent Authorities.

Since this study is blinded, the patient’s treatment code should be broken (procedure to be specified) before the reporting to the Competent Authorities and the Ethics Committees.

**13. DATA HANDLING AND MONITORING**

The Investigator assumes the responsibility to assure that the study will be conducted according to the protocol, the Good Clinical Practice (GCP) and ICH guidelines, local laws and obligations and the World Medical Association Declaration of Helsinki (Appendix 1) and that all valid data will be entered into the CRF.

Individual patient medical information obtained as a result of this study is considered confidential and disclosure to third part is prohibited.

The data must be recorded in the CRF, there should be an explanation for missing data, there should be no empty spaces, if a test is not performed, it should be written «NO», or the empty space should be barred. The data should be recorded with a black pen and in a legible way. In case of error, the error should be cancelled with a single line, the correct data should be written beside, and the correction should be dated and signed. If necessary the motivation for the correction should be indicated, data should never be obscured by use of corrective fluid or by overwriting.

The Investigator/s should prepare and store the lists of screened and randomised patients. For each one the following information should be included: identity, complete address, study number of patient, date of screening, date of enrolment and date of randomisation.

The Investigator/Institution will permit the representative of the Sponsor and the CRO to review in detail the CRF and the related data/original documents.

**14. STATISTICAL ISSUES**

Specification on statistical evaluation for efficacy and safety parameters

Continuous variables will be described using descriptive analysis like mean and median value, standard deviation, range, minimum and maximum; categorical variables will be described using relative and absolute frequencies.

Summary values will be expressed as mean ± SD. Categorical data will be compared by using Chi-square and the Fisher’s exact test. Normal distribution of variables will be estimated with Kolmogorov-Smirnov test. Normally distributed data will be compared using an independent t test. Skewed data will be analyzed non-parametrically (Mann-Whitney U or Kruskal-Wallis test). Serial measurements will be compared by repeated measures analysis of variance (RMANOVA). When significant, differences within and between each group will be tested by a post hoc analysis using the Bonferroni correction for multiple comparisons. A computerized statistical program (SPSS for Windows) will be used for all analyses. All tests will be 2-sided. The p values <0.05 will be considered statistically significant.

Survival analysis will be performed using clinical data obtained at follow-up (major and minor events). Kaplan-Meier method will be used to describe the overall survival and the length of the progression-free interval. Log-rank test will be used to compare survival curves. Univariate and multivariate analysis using the Cox regression model will be performed in order to eliminate eventually confounding factors.

## Interim analysis

Interim analyses will be programmed during the study. Interim analysis of data collected is programmed upon completion of follow-up of the first 15 and 30 patients to eventually discontinue the study in the case of excess adverse events or mortality.

In case of excess serious expected adverse drug reactions, deaths or SUSARs the Data Monitoring Committee will evaluate the results of the analysis in order to decide whether triiodothyronine treatment has provided proof “beyond reasonable doubt” that for all or for some specific type of patients, the trial treatment is clearly contraindicated in term of net difference in mortality and/or adverse drug reactions. Appropriate criteria of “proof beyond responsible doubt” cannot be specified precisely, but in general, a difference of at least 3 standard deviations in *ad interim* analysis of mortality and/or adverse drug reactions would be needed to justify halting or modifying the trial prematurely. In this case, the sponsor will discontinue the study and provide an analytical report to the competent authority within 4 weeks. During interim analysis, the Data Monitoring Committee is obliged to evaluate whether enrolled patients meet the inclusion and exclusion criteria according to the protocol, whether the protocol is being followed, the acceptability of data being accrued, the success of planned accrual targets and the appropriateness of the design assumptions.

Data Monitoring Committee is composed of the representative of the sponsor, Professor of Anaesthesiology Georgia Kostopanagiotou, Professor of Pharmacology Constantinos Pantos, Assistant Professor of Pharmacology Iordanis Mourouzis and Director and Cardiologist Athanasios Trikas.

## Criteria for the termination of the trial

Data Monitoring Committee will terminate the study before completion and provide an analytical report to the competent authority of each concerned Member State within 4 weeks in case of excess deterioration is recorded in response to treatment, such as deaths, excess serious expected adverse drug reactions, or SUSARs.

Under normal circumstances, the end of the trial will be the date of the last visit of the last patient participating in the trial.

## Procedure to account for missing or spurious data

All efforts will be directed towards minimizing the amount of missing data likely to occur. The percentage of missing values for a given variable in this study is expected not to exceed 5%.

Imputation of missing data is going to be performed based upon maximum-likelihood methodology. The maximum-likelihood based strategies fit a model by an iterative process (the Expectation maximisation algorithm). Multiple imputation methods generate multiple copies of the original dataset replacing missing values by randomly generated values and analyse them as complete sets.

Mixed effects models are proposed to be used to impute missing data of repeated measurements over time outcomes. Time of measurement may be considered as a random variable and these models may estimate a slope to summarize each patient’s response.

A sensitivity analysis will be performed at the end of the study in order to justify the choice of the particular method applied for handling missing data. Sensitivity analysis will consist of the following steps:

1. Comparison of the results of two analyses, one assigning the best possible outcome to all missing values in both groups and the other assigning the worst possible outcome to all missing values in both groups
2. Comparison of the results of two analyses, one assigning the best possible outcome to missing values in the control group and the worst possible to those of the experimental group and vice-versa.
3. Comparison of the results of the full set analysis to those of the complete case analysis

**15. ETHICAL ISSUES**

The study will be conducted according to the Good Clinical Practice (GCP) and ICH guidelines, local laws and obligations and the World Medical Association Declaration of Helsinki *(Appendix 1)*.

**15.1 Ethics Committees**

The protocol, the Patient information leaflet, the informed consent Form and the Investigator’s Brochure will be submitted to the relevant ***Ethics Committees***.

The written approval from the EC should clearly mention the approval of the protocol and the Patient information leaflet.

Any amendment to the protocol, before implementation, will be submitted to the relevant regulatory body for approval, after prior discussion between the Sponsor and the Co-ordinating Investigator.

**15.2 Informed consent**

Prior to being enrolled into the study, each patient or legal representativewill be required to give written informed consent. The Investigator will explain in writing the nature of the study, its objectives and potential risks. Each patient or legal representativewill have the opportunity to discuss the informed consent with the Investigator prior to give his/her written consent.

**15.3 Patient’s Insurance**

Sponsor holds and will maintain an adequate insurance policy covering damages arising out of Sponsor’s clinical research studies.

Sponsor will indemnify the Investigator and hold him/her harmless for claims for damages arising out of the investigation in excess of those covered by his/her own professional liability insurance providing that the drug was administered under his/her or deputy’s supervision and in strict accordance with accepted medical practice and the study protocol.

This indemnification does not apply to claims for damages arising out of any act of omission in his/her part or on the part of those under his/her supervision which shall or may amount to negligence in law.

The insurance policy should apply from the beginning until the end of the study.

The pharmaceutical company-sponsor is liable for any direct or indirect damage caused to the participant from the administration of the drug or any clinical intervention or procedure during participation in the study, which would not have taken place if the participant did not receive part in the study. All serious adverse events / reactions are covered and insured, according to Ministerial Decision DYG3 / 89292 / 31.12.2003.

The sponsor is exempted from that liability if the damage is due solely to the fault of the participant or failure to observe the instructions given.

If pregnancy occurs and the neonate experiences health problems due certainly to the investigational medicinal product, even though the protocol and consent form provided specific detailed information on the risks of the IMP and on prevention of pregnancy and although the study subject received all appropriate contraceptive measures, these cases are covered and secured as well.

The Investigator must notify to Sponsor immediately upon notice of any claims or lawsuits.

**16. USE OF INFORMATION**

The sponsor provides all study documents in confidence to the Investigator and his/her appointed staff. None of this material may be disclosed to any party not directly involved in the study without written permission from Sponsor.

Investigator must assure the patient’s anonymity will be maintained. The Investigator will keep a separate log of the patient’s study numbers, names, addresses and telephone numbers. The Investigator will maintain this for the longest period of time allowed from own institution and in any case, till further communication from Sponsor. The Investigator will supply the Sponsor with all the data/results from the study. All information concerning the study and the drug is confidential and the property of the Sponsor.

The Sponsor will prepare the final report, including the statistical and clinical evaluations. The Investigator’s agreement and signature will be obtained and a copy will be provided to the Investigator. Data Monitoring Committee will sign the final report. Sponsor reserves the right to publish and present the results of this study at scientific meetings, or to submit these clinical trial data to national and international Regulatory Authorities. The investigator may not use the results of this study for publication or presentation without authorization from Sponsor.

**17. RETENTION OF RECORD**

All documentation pertaining to the study will have to be kept by the Sponsor for the life time of the product. The final report, pertaining to this study, will have to be kept 5 years longer.

As required by GCP guidelines, the Investigator will have to keep the patient’s identification code, records and documents pertaining to the conduct of the study and the distribution of the investigational drug (i.e.: CRF, Consent Forms, drug accountability Forms and other pertinent information) for the longest possible duration, but at least for 15 years after the completion or discontinuation of the study.

No data should be destroyed without agreement with the Sponsor.

**18. REFERENCES**

1. Weiss P, Murdoch DR 2020 Clinical course and mortality risk of severe COVID-19. . Lancet:pii: S0140-6736(0120)30633-30634.

2. Yang X, Yu Y, Xu J, Shu H, Xia J, Liu H, Wu Y, Zhang L, Yu Z, Fang M, Yu T, Wang Y, Pan S, Zou X, Yuan S, Shang Y 2020 Clinical course and outcomes of critically ill patients with SARS-CoV-2 pneumonia in Wuhan, China: a single-centered,retrospective, observational study. Lancet Respir Med pii:S2213-2600(2220)30079-30075.

3. Cecconi M, Evans L, Levy M, Rhodes A 2018 Sepsis and septic shock. Lancet 392:75-87.

4. Trzeciak S, Cinel I, Phillip Dellinger R, Shapiro NI, Arnold RC, Parrillo JE, Hollenberg SM 2008 Microcirculatory Alterations in Resuscitation and Shock (MARS) Investigators. Resuscitating the microcirculation in sepsis: the central role of nitric oxide, emerging concepts for novel therapies, and challenges for clinical trials. Acad Emerg Med 15:399-413.

5. Vincent JL, De Backer D 2005 Microvascular dysfunction as a cause of organdysfunction in severe sepsis. Crit Care 9:S9-12.

6. Meineke R, Rimmelzwaan GF, Elbahesh H 2019 Influenza Virus Infections and Cellular Kinases. Viruses 11:pii: E171

7. Li R, Lin H, Ye Y, Xiao Y, Xu S, Wang J, Wang C, Zou Y, Shi M, Liang L, Xu H 2018 Attenuation of antimalarial agent hydroxychloroquine on TNF-α-induced endothelial inflammation. Int Immunopharmacol 63:261-269.

8. Shi Y, Fukuoka M, Li G, Liu Y, Chen M, Konviser M, Chen X, Opavsky MA, Liu PP 2010 Regulatory T cells protect mice against coxsackievirus-induced myocarditis through the transforming growth factor beta-coxsackie-adenovirus receptor pathway. Circulation 121:2624-2634.

9. Ma XL, Kumar S, Gao F, Louden CS, Lopez BL, Christopher TA, Wang C, Lee JC, Feuerstein GZ, Yue TL 1999 Inhibition of p38 mitogen-activated protein kinase decreases cardiomyocyte apoptosis and improves cardiac function after myocardial ischemia and reperfusion. Circulation 99:1685–1691

10. Pantos C, Mourouzis I, Saranteas T, Clave G, Ligeret H, Noack-Fraissignes P, Renard PY, Massonneau M, Perimenis P, Spanou D, Kostopanagiotou G, Cokkinos DV 2009 Thyroid hormone improves postischaemic recovery of function while limiting apoptosis: a new therapeutic approach to support hemodynamics in the setting of ischaemia-reperfusion? Basic Res Cardiol 104:69-77

11. Marchant D, Singhera GK, Utokaparch S, Hackett TL, Boyd JH, Luo Z, Si X, Dorscheid DR, McManus BM, Hegele RG 2010 Toll-like receptor 4-mediated activation of p38 mitogen-activated protein kinase is a determinant of respiratory virus entry and tropism. J Virol 84:11359-11373.

12. Celestino I, Checconi P, Amatore D, De Angelis M, Coluccio P, Dattilo R, Alunni Fegatelli D, Clemente AM, Matarrese P, Torcia MG, Mancinelli R, Mammola CL, Garaci E, Vestri AR, Malorni W, Palamara AT, Nencioni L 2018 Differential Redox State Contributes to Sex Disparities in the Response to Influenza Virus Infection in Male and Female Mice. Front Immunol 9:1747.

13. Pantos C, Tseti I, Mourouzis I 2020 Use of Triiodothyronine to treat critically ill COVID-19 patients: a new clinical trial. Crit Care 24:209

14. Dellinger RP, Levy MM, Rhodes A, Annane D, Gerlach H, Opal SM, Sevransky JE, Sprung CL, Douglas IS, Jaeschke R, Osborn TM, Nunnally ME, Townsend SR, Reinhart K, Kleinpell RM, Angus DC, Deutschman CS, Machado FR, Rubenfeld GD, Webb SA, Beale RJ, Vincent JL, Moreno R 2013 Surviving Sepsis Campaign Guidelines Committee including the Pediatric Subgroup. Surviving sepsis campaign: international guidelines for management of severe sepsis and septic shock: 2012. Crit Care Med 41:580-637.

15. Sato R, Ariyoshi N, Hasegawa D, Crossey E, Hamahata N, Ishihara T, Nasu M, Devendra G 2019 Effects of Inotropes on the Mortality in Patients With Septic Shock. J Intensive Care Med 885066619892218 (in press)

16. Léopold V, Gayat E, Pirracchio R, Spinar J, Parenica J, Tarvasmäki T, Lassus J, Harjola VP, Champion S, Zannad F, Valente S, Urban P, Chua HR, Bellomo R, Popovic B, Ouweneel DM, Henriques JPS, Simonis G, Lévy B, Kimmoun A, Gaudard P, Basir MB, Markota A, Adler C, Reuter H, Mebazaa A, Chouihed T 2018 Epinephrine and short-term survival in cardiogenic shock: an individual data meta-analysis of 2583 patients. Intensive Care Med 44:847-856.

17. Mourouzis I, Saranteas T, Ligeret H, Portal C, Perimenis P, Pantos C 2014 Phenylephrine postconditioning increases myocardial injury: are alpha-1 sympathomimetic agonist cardioprotective? Ann Card Anaesth 17:200-209

18. Marchant D, Dou Y, Luo H, Garmaroudi FS, McDonough JE, Si X, Walker E, Luo Z, Arner A, Hegele RG, Laher I, McManus BM 2009 Bosentan enhances viral load via endothelin-1 receptor type-A-mediated p38 mitogen-activated protein kinase activation while improving cardiac function during coxsackievirus-induced myocarditis. Circ Res 104:813-821.

19. Russell CD, Millar JE, Baillie JK 2020 Clinical evidence does not support corticosteroid treatment for 2019-nCoV lung injury. Lancet 395:473-475

20. Kim JG, Shin H, Kim W, Lim TH, Jang B, Cho Y, Choi KS, Ahn C, Lee J, Na MK 2018 The Value of Decreased Thyroid Hormone for Predicting Mortality in Adult Septic Patients: A Systematic Review and Meta-Analysis. Sci Rep 8:14137.

21. Padhi R, Kabi S, Panda BN, Jagati S 2018 Prognostic significance of nonthyroidal illness syndrome in critically ill adult patients with sepsis. Int J Crit Illn Inj Sci 8:165-172.

22. Zhao Y, Wang WY, Tian J, Zhang X, Yang M, Chen J, Mu M, Tang YD 2019 Impact of low T3 syndrome on adverse cardiovascular events in adult patients with acute viral myocarditis. Zhonghua Xin Xue Guan Bing Za Zhi 47:447-451.

23. Mourouzis I, Politi E, Pantos C 2013 Thyroid hormone and tissue repair: new tricks for an old hormone? J Thyroid Res 2013:312104

24. Pantos C, Mourouzis I, Cokkinos DV 2012 Thyroid hormone and cardiac repair/regeneration: from Prometheus myth to reality? Can J Physiol Pharmacol 90:977-987

25. Pantos C, Mourouzis I 2018 Thyroid hormone receptor α1 as a novel therapeutic target for tissue repair. Ann Transl Med 6:254

26. Pantos C, Mourouzis I, Saranteas T, Brozou V, Galanopoulos G, Kostopanagiotou G, Cokkinos DV 2011 Acute T3 treatment protects the heart against ischemia-reperfusion injury via TRalpha1 receptor. Mol Cell Biochem 353:235-241

27. Pantos C, Mourouzis I, Galanopoulos G, Gavra M, Perimenis P, Spanou D, Cokkinos DV 2010 Thyroid hormone receptor alpha1 downregulation in postischemic heart failure progression: the potential role of tissue hypothyroidism. Horm Metab Res 42:718-724

28. Bhargava M, Runyon MR, Smirnov D, Lei J, Groppoli TJ, Mariash CN, Wangensteen OD, Ingbar DH 2008 Triiodo-L-thyronine rapidly stimulates alveolar fluid clearance in normal and hyperoxia-injured lungs. Am J Respir Crit Care Med 178:506-512.

29. Hausenloy D, Chilian W, Crea F, Davidson S, Ferdinandy P, Garcia-Dorado D, van Royen N, Schulz R, Heusch G 2019 The coronary circulation in acute myocardial ischaemia/reperfusion injury—a target for cardioprotection. Cardiovasc Res 115:1143–1155.

30. Niccoli G, Montone R, Ibanez B, Thiele H, Crea F, Heusch G, Bulluck H, Hausenloy DJ, Berry C, Stiermaier T, Camici P, Eitel I 2019 Optimized treatment of ST-elevation myocardial infarction: the unmet need to target coronary microvascular obstruction as primary treatment goal to further improve prognosis. Circ Res 125:245–258.

31. Elgendy I, Jneid H 2018 Microvascular obstruction in ST elevation myocardial infarction patients undergoing primary percutaneous coronary intervention: another frontier to conquer? J Thorac Dis 10:1343-1346.

32. de Waha S, Patel M, Granger C, Ohman E, Maehara A, Eitel I, Ben-Yehuda O, Jenkins P, Thiele H, Stone G 2017 Relationship between microvascular obstruction and adverse events following primary percutaneous coronary intervention for ST-segment elevation myocardial infarction: an individual patient data pooled analysis from seven randomized trials. Eur Heart J 38:3502-3510.

33. Pantos C, Mourouzis I L-triiodothyronine (T3) for use in limiting microvascular obstruction. PCT/EP2019/087056:(pending patent)

34. Pantos C, Mourouzis I Composition comprising L-triiodothyronine (T3) for use in the treatment of critically ill patients with coronavirus infection. Greek Patent Office number of case:22-0002577373:(pending patent)

35. Figliozzi RW, Chen F, Hsia SV 2017 New insights on thyroid hormone mediated regulation of herpesvirus infections. Cell Biosci 7:13

36. Varedi M, Moattari A, Amirghofran Z, Karamizadeh Z, Feizi H 2014 Effects of hypo- and hyperthyroid states on herpes simplex virus infectivity in the rat. Endocr Res 39:50-55.

37. Varedi M, Shiri H, Moattari A, Omrani GH, Amirghofran Z 2014 Hyperthyroid state or in vitro thyroxine treatment modulates TH1/TH2 responses during exposure to HSV-1 antigens. J Immunotoxicol 11:160-165.

38. Hsia SV, Chen LH, Tseng HF 2017 Receipt of thyroid hormone deficiency treatment and risk of herpes zoster. Int J Infect Dis 59:90-95.

39. Hodkinson CF, Simpson EE, Beattie JH, O'Connor JM, Campbell DJ, Strain JJ, Wallace JM 2009 Preliminary evidence of immune function modulation by thyroid hormones in healthy men and women aged 55-70 years. J Endocrinol 202:55-63.

40. Strich D, Karavani G, Edri S, Chay C, Gillis D 2017 FT3 IS HIGHER IN MALES THAN IN FEMALES AND DECREASES OVER THE LIFESPAN. Endocr Pract 23:803-807.

41. Strich D, Karavani G, Edri S, Gillis D 2016 TSH enhancement of FT4 to FT3 conversion is age dependent. Eur J Endocrinol 175:49-54.

42. Provinciali M, Muzzioli M, Di Stefano G, Fabris N 1991 Recovery of spleen cell natural killer activity by thyroid hormone treatment in old mice. Nat Immun Cell Growth Regul 10:226-236.

43. Botella-Carretero JI, Prados A, Manzano L, Montero MT, Escribano L, Sancho J, Escobar-Morreale HF 2005 The effects of thyroid hormones on circulating markers of cell-mediated immune response, as studied in patients with differentiated thyroid carcinoma before and during thyroxine withdrawal. Eur J Endocrinol 153:223-230.

44. Mason JW, O'Connell JB, Herskowitz A, Rose NR, McManus BM, Billingham ME, Moon TE 1995 A clinical trial of immunosuppressive therapy for myocarditis. The Myocarditis Treatment Trial Investigators. N Engl J Med 333:269-275.

45. Paniz-Mondolfi A, Bryce C, Grimes Z, Gordon RE, Reidy J, Lednicky J, Sordillo EM, Fowkes M 2020 Central Nervous System Involvement by Severe Acute Respiratory Syndrome Coronavirus -2 (SARS-CoV-2). J Med Virol:(in press)

46. Fan E, Brodie D, Slutsky AS 2018 Acute Respiratory Distress Syndrome: Advances in Diagnosis and Treatment. JAMA 319:698-710.

47. Munshi L, Del Sorbo L, Adhikari NKJ, Hodgson CL, Wunsch H, Meade MO, Uleryk E, Mancebo J, Pesenti A, Ranieri VM, Fan E 2017 Prone Position for Acute Respiratory Distress Syndrome. A Systematic Review and Meta-Analysis. Ann Am Thorac Soc 14:S280-S288.

48. Hesch RD, Hüsch M, Ködding R, Höffken B, Meyer T 1981 Treatment of dopamine-dependent shock with triiodothyronine. Endocr Res Commun 8:229-237.

**APPENDICES**

**Appendix1**: Declaration of Helsinki

**Appendix 2**: Investigator’s approval Page

**APPENDIX 1**

**The World Medical Association Declaration of Helsinki**

### *World Medical Association Declaration of Helsinki: Recommendations Guiding Medical Doctors in Biomedical Research Involving Human Subjects*

#### Adopted by the 18^°^World Medical Assembly, Helsinki, Finland, 1964 and as revised by the World Medical Assembly in Tokyo, Japan in 1975, in Venice, Italy in 1983, and in Hong Kong in 1989.

**Introduction**

It is the mission of the physician to safeguard the health of the people. His or her knowledge and conscience are dedicated to the fulfillment of this mission.

The [Declaration of Geneva](http://www.cirp.org/library/ethics/geneva/) of the World Medical Association binds the physician with the words, "The health of my patient will be my first consideration," and the International Code of Medical Ethics declares that, "A physician shall act only in the patient's interest when providing medical care which might have the effect of weakening the physical and mental condition of the patient."

The Purpose of biomedical research involving human subjects must be to improve diagnostic, therapeutic and prophylactic procedures and the understanding of the aetiology and pathogenesis of disease.

In current medical practice most diagnostic, therapeutic or prophylactic procedures involve hazards. This applies especially to biomedical research.

Medical progress is based on research which ultimately must rest in part on experimentation involving human subjects.

In the field of biomedical research a fundamental distinction must be recognized between medical research in which the aim is essentially diagnostic or therapeutic for a patient, and medical research, the essential object of which is purely scientific and without implying direct diagnostic or therapeutic value to the person subjected to the research.

Special caution must be exercised in the conduct of research which may affect the environment, and the welfare of animals used for research must be respected.

Because it is essential that the results of laboratory experiments be applied to human beings to further scientific knowledge and to help suffering humanity, the World Medical Association has prepared the following recommendations as a guide to every physician in biomedical research involving human subjects. They should be kept under review in the future. It must be stressed that the standards as drafted are only a guide to physicians all over the world. Physicians are not relieved from criminal, civil and ethical responsibilities under the laws of their own countries.

**I. Basic Principles**

1. Biomedical research involving human subjects must conform to generally accepted scientific principles and should be based on adequately performed laboratory and animal experimentation and on a thorough knowledge of the scientific literature.
2. The design and performance of each experimental procedure involving human subjects should be clearly formulated in an experimental protocol which should be transmitted for consideration, comment and guidance to a specially appointed committee independent of the investigator and the sponsor provided that this independent committee is in conformity with the laws and regulations of the country in which the research experiment is performed.
3. Biomedical research involving human subjects should be conducted only by scientifically qualified persons and under the supervision of a clinically competent medical person. The responsibility for the human subject must always rest with a medically qualified person and never rest on the subject of the research, even though the subject has given his or her consent.
4. Biomedical research involving human subjects cannot legitimately be carried out unless the importance of the objective is in proportion to the inherent risk to the subject.
5. Every biomedical research project involving human subjects should be preceded by careful assessment of predictable risks in comparison with foreseeable benefits to the subject or to others. Concern for the interests of the subject must always prevail over the interests of science and society.
6. The right of the research subject to safeguard his or her integrity must always be respected. Every precaution should be taken to respect the privacy of the subject and to minimize the impact of the study on the subject's physical and mental integrity and on the personality of the subject.
7. Physicians should abstain from engaging in research projects involving human subjects unless they are satisfied that the hazards involved are believed to be predictable. Physicians should cease any investigation if the hazards are found to outweigh the potential benefits.
8. In publication of the results of his or her research, the physician is obliged to preserve the accuracy of the results. Reports of experimentation not in accordance with the principles laid down in this Declaration should not be accepted for publication.
9. In any research on human beings, each potential subject must be adequately informed of the aims, methods, anticipated benefits and potential hazards of the study and the discomfort it may entail. He or she should be informed that he or she is at liberty to abstain from participation in the study and that he or she is free to withdraw his or her consent to participation at any time. The physician should then obtain the subject's freely-given informed consent, preferably in writing.
10. When obtaining informed consent for the research project the physician should be particularly cautious if the subject is in a dependent relationship to him or her or may consent under duress. In that case the informed consent should be obtained by a physician who is not engaged in the investigation and who is completely independent of this official relationship.
11. In case of legal incompetence, informed consent should be obtained from the legal guardian in accordance with national legislation. Where physical or mental incapacity makes it impossible to obtain informed consent, or when the subject is a minor, permission from the responsible relative replaces that of the subject in accordance with national legislation. Whenever the minor child is in fact able to give a consent, the minor's consent must be obtained in addition to the consent of the minor's legal guardian.
12. The research protocol should always contain a statement of the ethical considerations involved and should indicate that the principles enunciated in the present Declaration are complied with.

**II. Medical Research Combined with Professional Care (Clinical Research)**

1. In the treatment of the sick person, the physician must be free to use a new diagnostic and therapeutic measure, if in his or her judgment it offers hope of saving life, reestablishing health or alleviating suffering.
2. The potential benefits, hazards and discomfort of a new method should be weighed against the advantages of the best current diagnostic and therapeutic methods.
3. In any medical study, every patient--including those of a control group, if any--should be assured of the best proven diagnostic and therapeutic method.
4. The refusal of the patient to participate in a study must never interfere with the physician-patient relationship.
5. If the physician considers it essential not to obtain informed consent, the specific reasons for this proposal should be stated in the experimental protocol for transmission to the independent committee (I,2).
6. The physician can combine medical research with professional care, the objective being the acquisition of new medical knowledge, only to the extent that medical research is justified by its potential diagnostic or therapeutic value for the patient.

**III. Non-Therapeutic Biomedical Research Involving Human Subjects (Non-Clinical Biomedical Research)**

1. In the purely scientific application of medical research carried out on a human being, it is the duty of the physician to remain the protector of the life and health of that person on whom biomedical research is being carried out.
2. The subjects should be volunteers--either healthy persons or patients for whom the experimental design is not related to the patient's illness.
3. The investigator or the investigating team should discontinue the research if in his/her or their judgment it may, if continued, be harmful to the individual.
4. In research on man, the interest of science and society should never take precedence over considerations related to the well-being of the subject.

**INVESTIGATOR’S APPROVAL PAGE**

***This page will be signed by the Principal Investigator, the Coordinator and the Sponsor’s Medical Expert, in two protocols.***

***The first one will be kept in the Sponsor’s trial master file and the second one in the Centre file. The present will be submitted to the Competent Authorities.***

I, the undersigned, representing the sponsor of the study, I have studied the protocol version 3 dated 11/05/2020, of the protocol T3inj-02 entitled “Triiodothyronine for the treatment of critically ill patients with COVID-19 infection (The Thy-**SUPPORT** study)” and confirm that the protocol version 3 dated 11/05/2020 adheres to the guidelines of Good Clinical Practice (ICH GCP guidelines).

|  |  |  |
| --- | --- | --- |
| Name |  |  |
|  |  |  |
| Signature |  | Date |

I, the undersigned, principal investigator/coordinator, I have studied the protocol version 3 dated 11/05/2020, of the protocol T3inj-02 entitled “Triiodothyronine for the treatment of critically ill patients with COVID-19 infection (The Thy**SUPPORT** study)”. I confirm that all my collaborators who will participate in the conduct of this study are aware of their obligations.

I confirm that my study site has all necessary facilities to collect all necessary information during the conduct of this study.

I confirm that I will conduct the study according to the study protocol, current guidelines of Good Clinical Practice (ICH GCP guidelines), and according to the Greek law.

|  |  |  |
| --- | --- | --- |
| Name |  |  |
|  |  |  |
| Signature |  | Date |
